# Cyclopropanation–ring expansion of 3-chloroindoles with α-halodiazoacetates: novel synthesis of 4-quinolone-3-carboxylic acid and norfloxacin

**DOI:** 10.3762/bjoc.15.212

**Published:** 2019-09-13

**Authors:** Sara Peeters, Linn Neerbye Berntsen, Pål Rongved, Tore Bonge-Hansen

**Affiliations:** 1 Department of Chemistry, University of Oslo, P.O. Box 1033 Blindern, NO-0315 Oslo, Norway; 2 Department of Pharmacy, University of Oslo, P.O. Box 1068 Blindern, NO-0316, Oslo, Norway

**Keywords:** catalysis, cyclopropanation, indole, norfloxacin quinoline, quinolone, Rh(II), ring expansion

## Abstract

We present a short and efficient way of synthesizing two synthetically versatile 4-quinolone-3-carboxylate building blocks by cyclopropanation-ring expansion of 3-chloroindoles with α-halodiazoacetates as the key step. This novel transformation was applied towards the synthesis of the antibiotic drug norfloxacin.

## Introduction

The development and use of metal carbenes occupy a central part in the field of the C–H functionalization [1]. Among the metal carbenes, the transient Rh carbenes, usually made by Rh-catalyzed decomposition of diazo compounds, are particularly versatile intermediates in organic synthesis, as they partake in cyclopropanation and C–H insertion reactions with high levels of selectivity [2]. This transition-metal-catalyzed carbene transfer has emerged as a mild and attractive route to indole functionalization [3–6]. The metal carbene reactions with indoles have been studied for the three types of carbenoids: acceptor–acceptor [7–9], mono-acceptor [10] and donor–acceptor carbenoids [11–14]. Depending on the metal and the diazo compound, the chemo- and regioselectivity in the metal carbene transfer reaction typically give N–H, C–H (at C3) and double N–H/C–H insertion products. The presence of electron-withdrawing groups on the indole nitrogen makes it possible to cyclopropanate the indole C2–C3 double bond and isolate indoline cyclopropanes.

**Scheme 1:**
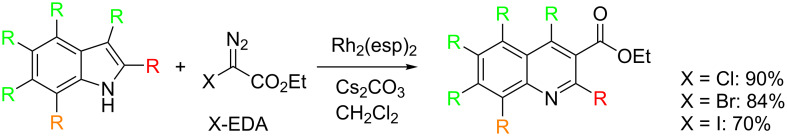
The effect of indole substituents on the yields of ethyl quinoline-3-carboxylates [15]. Green = good, orange = medium, red = poor. R = halogen, Me, MeO, NO_2_.

We recently discovered that Rh carbenes derived from ethyl α-halodiazoacetates (X-EDA) react readily with unprotected indoles to form ethyl 3-carboxyquinoline structures (Scheme 1) [15].

The Rh carbenes derived from X-EDAs stand out from the three other types of classified carbenoids with respect to chemoselectivity in reactions with indoles. These halo-acceptor carbenoids undergo cyclopropanation of N–H indoles with high selectivity, and only traces of C–H or N–H insertion products were observed. The yield of ethyl quinoline-3-carboxylate is dependent on the halogen in X-EDA (Cl: 90%, Br: 84%, I: 70%). The reaction works well for substituted indoles with R-groups in positions 3–7. When R at position 8 = Cl, the reaction was sluggish, and when R at position 2 = Me there was no quinoline formed. The overall transformation is formally a cyclopropanation of the indole C2–C3 double bond followed by a spontaneous ring expansion of the indoline cyclopropane intermediate and elimination of HX.

**Figure 1:**
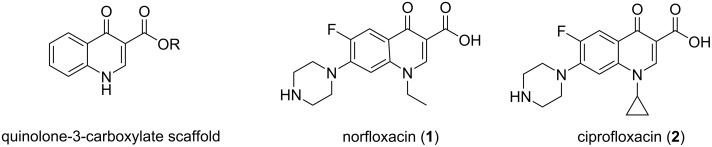
Quinolone 3-carboxylate scaffold, norfloxacin (**1**) and ciprofloxacin (**2**).

The 4-quinolone-3-carboxylic acid scaffold (Figure 1) is regarded as a privileged scaffold in medicinal chemistry, due to the frequent appearance of this structural subunit in many commercial drugs [16–21], and a large variety of compounds with biological activities ranging from antitumor, antiviral, antibiotic and antiparasitic to cannabinoid receptor modulating [22–28].

Despite the attractive features of the quinolone-3-carboxylate scaffold, there are only four reported routes towards this valuable structural unit. The common denominator for three of the routes (Grohe and Heitzer [29], Gould and Jacobs [30] and Long [31]) is that they consist of ring-closure reactions to form the quinolone ring system. The fourth one (Reddy) utilizes oxidative ring cleavage of the indole ring followed by condensation [32]. Some of these routes suffer from harsh reactions conditions and there is clearly room for novel methods complementary to those already present.

Heindel and Fine reported back in 1970 that 4-(1*H*)-quinolones can be synthesized in a straightforward fashion by simply heating 4-chloroquinolines in ethanol [33]. This transformation is particularly well suited for 4-chloroquinolines with electron withdrawing substituents (ester and nitro) in the 3-position. Both the initial displacement of the 4-chloro-substituent and the cleavage of the 4-alkoxyquinoline is auto-catalyzed and takes place under neutral conditions without the need for external acid catalysis. These relatively mild reaction conditions should be applicable to a large substrate scope.

**Scheme 2:**
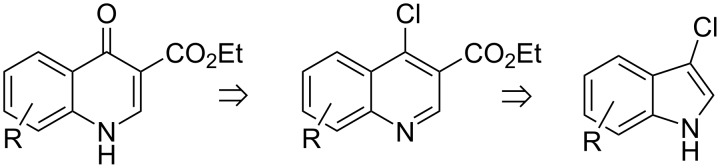
Retrosynthetic outline for the synthesis of quinolone-3-carboxlates from indole derivatives.

We anticipated that our novel cyclopropanation–ring expansion reaction would apply to 3-chloroindol derivatives, and designed a simple and straightforward retrosynthetic approach towards quinolone-3-carboxylates, as outlined in Scheme 2.

Cyclopropanation–ring expansion of 3-chloroindole derivatives with X-EDA would give ethyl 4-chloro-quinoline-3-carboxylates, which would subsequently undergo alcoholysis to give 4-quinolone-3-carboxylates.

## Results and Discussion

**Scheme 3:**
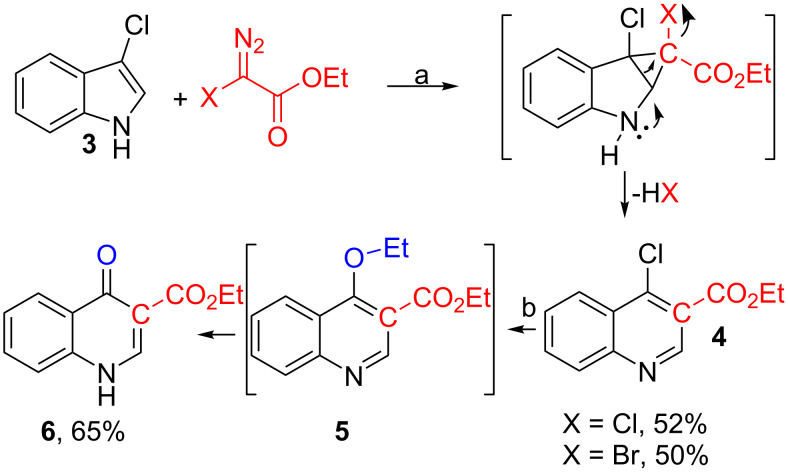
Synthesis of ethyl 4-quinolone-3-carboxylate (**6**) and proposed mechanism. a: Rh_2_(esp)_2_ (1 mol %), CH_2_Cl_2_, rt, Cs_2_CO_3_, 50–52%. b: EtOH, reflux, 24 h, 65%.

We started with the simplest 3-chloroindole available (Scheme 3).

The choice of reaction conditions for the cyclopropanation–ring expansion of 3-chloroindole (**3**) with X-EDA was based on our previous work (Scheme 1) [15]. During our investigation of the cyclopropanation–ring expansion of indoles with halodiazoacetates, we identified Rh_2_(esp)_2_ (1 mol %) as the optimal catalyst. Even though several other dirhodium(II)-catalysts could be used for this transformation, the yields were inferior compared to Rh_2_(esp)_2_. The choice of solvent was out of convenience. α-Halodiazoacetates are synthesized and quickly purified using CH_2_Cl_2_ as solvent, and solutions of X-EDA in CH_2_Cl_2_ are stable for hours and conveniently handled at 0 °C. In order to ease the chromatographic purification of the ethyl 3-carboxyquinoline products, we found it useful to use a slight excess (1.1–1.3 equiv) of X-EDA relative to the indole substrates (1.0 equiv). When we applied these standard conditions to the cyclopropanation–ring expansion of 3-chloroindole (**3**) with Cl-EDA or Br-EDA, we obtained ethyl 4-chloroquinoline carboxylate (**4**) in 50% and 52% yield, respectively. The presumed mechanism for this transformation is shown in Scheme 3. It starts with a cyclopropanation of the indole C2–C3 double bond to form an unstable, non-isolatable indoline cyclopropane carboxylate intermediate. This intermediate is nicely set up for a spontaneous ring expansion and elimination of HX to form **4**. After exposing **4** to refluxing ethanol, the desired ethyl 4-quinolone-3-carboxylate (**6**) precipitated in 65% yield. The quinoline **4** to quinolone **6** transformation is a two-step process [33]. The 4-chloro substituent is displaced by ethanol giving ethyl 4-ethoxy-quinoline-3-carboxylate (**5**, not isolated). Subsequent attack by ethanol or Cl^−^ cleaves off the ethyl group and yields quinolone (**6**) after tautomerization. Standard hydrolysis (LiOH, H_2_O/THF) of ester **6** gave the 4-quinolone-3-carboxylic acid (**7**) in 87% yield (not shown).

**Scheme 4:**
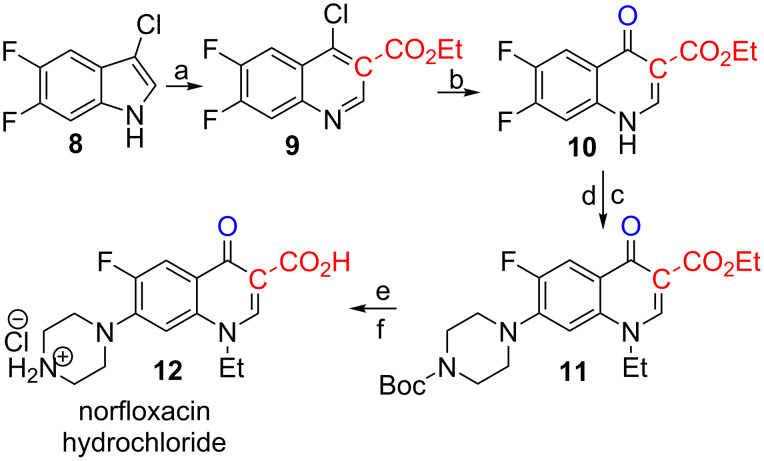
Synthesis of norfloxacin. a: Cl-EDA (1.3 equiv), Rh_2_(esp)_2_ (1 mol %), toluene, rt, Cs_2_CO_3_, 75%. b: EtOH, reflux, 24 h, 90%. c: EtI, K_2_CO_3_, DMF, 90 °C, 24 h, 85%. d: Boc-piperazine, K_2_CO_3_, CH_3_CN, reflux, 3 d, 70%. e: LiOH, MeOH/H_2_O, 20 h, 96%. f: TFA, DCM then 1 M HCl, 99%.

Quinolones, and fluoroquinolones (FQs) in particular, are among the most commonly prescribed antibiotics in the world [34]. Norfloxacin (**1**) is considered to be the first broad band antibiotic and its structurally related cousin ciprofloxacin (**2**) is one of the most prescribed antibiotics even after 20 years of clinical use. We elected **1** as a synthetic target for our novel approach towards FQs (Scheme 4). Chlorination of commercially available 6,7-difluoroindole gave the required substrate **8**.

The cyclopropanation–ring expansion reaction of **8** with Br-EDA in CH_2_Cl_2_ using Rh_2_(esp)_2_ as catalyst gave the 4-chloroquinoline-derivative **9** in 51% yield. The somewhat modest yield of **9** was similar to the yield of **4**, but was lower compared the general yields of 3-carboxy-quinolines obtained in our initial study. Two previous observations in our lab, one made in our cyclopropanation–ring expansion study of indoles, and one made during our initial reactions of X-EDAs with olefins [35], gave us the clues for how to quickly improve the yield of **9**. Cl-EDA gave a small, but significant increase in yield compared to Br-EDA in our previous study (90% vs 84% yield, Scheme 1). The cyclopropanations of olefins with X-EDAs were very sensitive to the choice of solvent, and the yields increased dramatically on going from CH_2_Cl_2_ to toluene. When we applied the combination of Cl-EDA as the X-EDA source and toluene as solvent, we obtained **9** in 75% yield. The transformation of quinoline **9** to quinolone **10** was very simple to execute experimentally. The 4-quinolone ethyl ester **10** precipitated in 90% yield after exposing **9** to refluxing ethanol for 24 h. When working on this transformation, we managed to isolate trace quantities of the 4-ethoxy-quinoline derivate (not shown) of **9**, hereby supporting the two-step process proposed by Heindel and Fine [33]. The *N*-ethyl group was introduced with Et-I under weakly basic conditions in 85% yield [36]. We initially introduced the piperazine unit in a quantitative yield with unprotected piperazine (not shown) [37–38]. However, purification and isolation were problematic and we found it more convenient to use Boc-piperazine (70% yield) to obtain **11**. Hydrolysis of the ethyl ester (96%) with LiOH and acidic removal of the Boc group (99%) gave norfloxacin as the hydrochloride salt (**12**) in an overall 35% yield from **8**.

## Conclusion

We have developed a novel and efficient synthetic route towards two privileged 4-quinolone-3-carboxylate scaffolds commonly used in medicinal chemistry. The highly attractive 6,7-difluoro-4-quinolone-3-carboxylate building block **10** was accessed in high yield in only three steps from commercially available substrates. The nature of the cyclopropanation–ring expansion makes the 4-chloro-3-carboxy-quinoline structure with perfect positioning of the carboxylate and chlorine substituents for the subsequent reaction to form the quinolone. The current work shows that the key cyclopropanation–ring expansion reaction works well with 3-chloroindole and 6,7-difluoro-3-chloro-indole, consistent with our previous work where substituents in positions 3–7 are well tolerated.

## Experimental

Detailed experimental procedures and analytical data for the compounds are available in Supporting Information File 1.

### General procedure for the synthesis of ethyl 4-chloroquinoline-3-carboxylates from X-EDA and 3-chloroindoles

X-EDA was synthesized according to a literature procedure [35]. This gave X-EDA in a cooled DCM solution (between 1.0 and 1.5 equiv). When desired, the solvent was swopped from DCM to toluene at 0 °C. The cooled X-EDA solution was transferred to an ice-cooled addition funnel (0 °C) and added slowly dropwise to a stirring solution of the desired 3-chloroindole (1.0 equiv), Cs_2_CO_3_ (1.3 equiv) and Rh_2_(esp)_2_ (0.01 equiv) in DCM or toluene at ambient temperature. Upon addition, the solution color changed from green/purple to orange/brown. Addition time was around 30–60 min. After all X-EDA was added, the solution was stirred for 30 min before the solvent was evaporated in vacuo. The crude product was dissolved in 30 mL EtOAc and washed with 3 mL H_2_O and 3 mL saturated NaCl solution. The organic phase was dried with MgSO_4_, filtered and the solvent evaporated in vacuo. The residue was purified using silica gel column chromatography.

### General procedure for alcoholysis of ethyl 4-chloroquinoline-3-carboxylates to ethyl 4(1*H*)-oxoquinolone-3-carboxylates [33]

To the desired ethyl 4-chloro-quinoline-3-carboxylate derivative (1–2 mmol) was added 5 mL dry ethanol. This solution was refluxed for 24 h or monitored by TLC analysis. The reaction mixture was cooled after the starting material was fully consumed (judged by TLC) and gave a white precipitation. The white solid was centrifuged, the liquid carefully removed and the solid was washed with EtOAc and centrifuged 2–3 times to leave a pure off-white solid.

## Supporting Information

File 1 Synthetic procedures, spectroscopic data and copies of NMR spectra.
