# Supplementary material for: Cyclopropanation–ring expansion of 3-chloroindoles with α-halodiazoacetates: novel synthesis of 4-quinolone-3-carboxylic acid and norfloxacin
Source: Beilstein J Org Chem. 2019 Sep 13;15:2156–60. doi: 10.3762/bjoc.15.212 (PMC6753669; doi:10.3762/bjoc.15.212)
Supplement: File 1 — Synthetic procedures, spectroscopic data and copies of NMR spectra. [file Beilstein_J_Org_Chem-15-2156-s001.pdf]

BEILSTEIN JOURNAL OF ORGANIC CHEMISTRY

Supporting Information

for

Cyclopropanation–ring expansion of 3-chloroindoles with α-halodiazoacetates: novel synthesis of 4-quinolone-3-carboxylic acid and norfloxacin

Sara Peeters, Linn Neerbye Berntsen, Pål Rongved and Tore Bonge-Hansen

Beilstein J. Org. Chem. **2019,** *15,* 2156–2160. doi:10.3762/bjoc.15.212

Synthetic procedures, spectroscopic data and copies of NMR spectra

License and Terms: This is a supporting information file under the terms of the Creative Commons Attribution License (http://creativecommons.org/licenses/by/4.0). Please note that the reuse, redistribution and reproduction in particular requires that the authors and source are credited.
The license is subject to the *Beilstein Journal of Organic Chemistry* terms and conditions: (https://www.beilstein-journals.org/bjoc)

Table of contens

General information	S2
Synthetic procedures and spectroscopic data	S3–S13
^{1}H and ^{13}C NMR spectra	S14–S23
References	S25

General Information

Solvents and chemicals were used as delivered from Sigma-Aldrich, VWR International AS and AK Scientific, unless stated otherwise. DCM, DMF and acetonitrile were dried using the MB SPS-800 solvent purification system from MBraun. Eluents used during flash chromatography were of technical grade. Technical grade hexane was distilled before used. NMR-solvents were used as delivered from Sigma-Aldrich and Cambridge Isotope Laboratories.

N-Chlorosuccinimide and *N*-bromosuccinimide were recrystallized before use.

Ethyl diazoacetate (EDA) – provided by Sigma Aldrich – contained ≥13 wt % DCM according to the supplier. ^{1}H NMR analysis of EDA in $CDCl_3$ gave around 9 wt % DCM. In all reactions where EDA is used as reactant, the 9 wt % DCM was taken into account when calculating the yields.

Thin layer chromatography (TLC) was performed on 60 F254 silica coated alumina plates from Merck. Flash Chromatography was performed on silica gel from Merck (Silicagel 60, 40–0.60 µm, 460–520 m^2/g, pH 6.5–7.5) either manually or with an Isco Inc. CombiFlash Companion with PeakTrack software (v.1.4.10).

^{1}H, ^{13}C and ^{19}F NMR experiments were recorded in $CDCl_3$, methanol-d_4, acetone-d_6, TFA-*d*, DMSO-d_6 or acetonitrile-d_3 using either a Bruker Avance DPX200, DPX300, AVII400, AVIII400, DRX500 or AVII600 instrument with residual peaks as references ($CDCl_3$: 7.24 ppm/77.0 ppm, methanol-d_4: 3.31 ppm/49.15 ppm, acetone-d_6: 2.05 ppm/206.7 ppm, CF_3COOD: 11.5 ppm/164.2 ppm, DMSO-d_6: 2.50 ppm/39.5 ppm or acetonitrile-d_3: 1.94 ppm/118.7 ppm). Chemical shifts (δ) are given in parts per million (ppm) and coupling constants (J) are given in Hertz (Hz). Multiplicities are abbreviated as: s – singlet; bs – broad singlet; d – doublet; t – triplet; q – quartet; dd – double doublet; ddd – doublet of doublets of doublets; dt – doublet of triplets; m – multiplet.

For new compounds, DEPT135, COSY and HSQC were recorded to assign ^{1}H and ^{13}C-shift.

Mass spectra were obtained on Bruker Daltonik GmbH MAXIS II ETD (ESI) and SCION-TQ (EI) spectrometer by Osamu Sekiguchi. Melting points were measured on Büchi B-545-melting point instrument.

Synthetic procedures and spectroscopic data

Cl-EDA and Br-EDA were synthesized from EDA according to a literature procedure.[1] We performed quantitative ^{1}H-NMR measurements and found the average yields of Cl-EDA and Br-EDA to be 94%.

3-Cl-indole and 3-Cl-6,7-difluoro-indole were synthesized from commercially available indole and 6,7-difluoro-indole according to a literature procedure.[2]

General procedure for the synthesis of ethyl 4-chloroquinoline-3-carboxylates from X-EDA and 3-chlororindoles.

X-EDA was synthesized according to a literature procedure.[1] This gave X-EDA in a cooled DCM solution (between 1.0 and 1.5 eqiuv). When desired, the solvent was swopped from DCM to toluene at 0 °C. The cooled X-EDA solution was transferred to an ice-cooled addition funnel (0 °C) and added slowly dropwise to a stirring solution of the desired 3-Cl-indole (1.0 equiv), Cs_2CO_3 (1.3 equiv) and $Rh_2(esp)_2$ (0.01 equiv) in DCM or toluene at ambient temperature. Upon addition, the solution color changed from green/purple to orange/brown. Addition time was around 30–60 min. After all X-EDA was added, the solution was stirred for 30 min. before the solvent was evaporated in vacuo. The crude product was dissolved in 30 mL EtOAc and washed with 3 mL H_2O and 3 mL saturated NaCl solution. The organic phase was dried with $MgSO_4$, filtered and the solvent evaporated in vacuo. The residue was purified using silica gel column chromatography.

General procedure for alcoholysis of ethyl 4-chloroquinoline-3-carboxylates to ethyl 4(1*H*)-oxo-quinolone-3-carboxylates.[3]

To the desired ethyl 4-chloro-quinoline-3-carboxylate derivative (1 – 2 mmol) was added 5 mL dry ethanol. This solution was refluxed for 24 h or monitored by TLC analysis. The reaction mixture was cooled after the starting material was fully consumed (judged by TLC) and gave a white precipitation. The white solid was centrifuged, the liquid carefully removed and the solid was washed with EtOAc and centrifuged 2–3 times to leave a pure off-white solid.

General procedure for hydrolysis of ethyl 4-oxoquinolone-3-carboxylates to 4-oxoquinolone-3-carboxylic acids.

The ethyl 4(1*H*)-oxo-quinolone-3-carboxylate (1–2 mmol) was dissolved in 5 mL MeOH. $LiOH \cdot H_2O$ (3.0 equiv), dissolved in 1–2 mL H_2O, was added to the reaction mixture. The reaction mixture was stirred at ambient temperature overnight or monitored by TLC analysis. After the starting material was fully consumed (judged by TLC-analysis), the reaction volume was reduced to one third of its initial volume. The solution was acidified to pH 2–3 (pH-paper) with 1 M HCl. The resulting white solution was centrifuged and the liquid carefully removed. The remaining solid was washed with water and centrifuged twice, leaving a pure off-white solid.

Synthesis of ethyl 4-chloroquinoline-3-carboxylate (4)

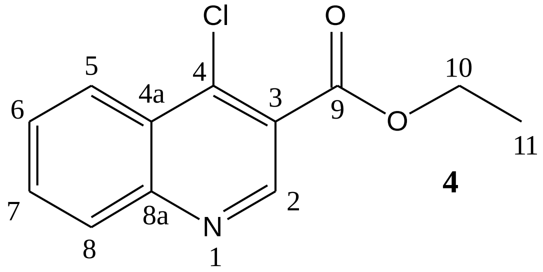

4

Ethyl 4-chloroquinoline-3-carboxylate (**4**) was synthesized according to the general procedure.

Yield: 52% with Br-EDA; 50% with Cl-EDA.

TLC (silica plates; eluent EtOAc:DCM:Hexane 1:8:1): R_f = 0.42

^{1}H NMR (400 MHz, $CDCl_3$): δ 9.15 ppm (s, 1H, H-2), 8.33 ppm (dd, *J* = 1.5 Hz; 1.3 Hz, 1H, H-5), 8.03 - 8.12 ppm (m, 1H, H-8), 7.78 ppm (ddd, *J* = 8.4 Hz; 6.9 Hz; 1.4 Hz, 1H, H-6), 7.63 ppm (ddd, *J* = 8.4 Hz; 6.9 Hz; 1.3 Hz, 1H, H-7), 4.45 ppm (q, *J* = 7.2 Hz, 2H, H-10), 1.42 ppm (t, *J* = 7.1 Hz, 3H, H-11)

^{13}C NMR (100 MHz, $CDCl_3$): δ 164.4 ppm (C-9), 149.9 ppm (C-4), 149.4 ppm (C-8a), 143.3 ppm (C-2), 131.7 ppm (C-7), 129.7 ppm (C-8), 128.2 ppm (C-6), 126.0 ppm (C-5), 125.3 ppm (C-4a), 122.8 ppm (C-3), 61.9 ppm (C-10), 14.2 ppm (C-11)

MS (EI, Methanol); m/z (relative intensity): 235 (M^+, 44%), 237 (M^+ + 2), 207 (35%), 209 (11%), 190 (100%), 192 (35%), 162 (41%), 164 (14%), 127 (19%)

HR-MS (ESI, Methanol): 258.0293 [M + Na^+]; calculated value for $C_{12}C_{10}ClNNaO_2$: 258.0292 (Δ -0.2 ppm); 260.0264 [M + Na^+ + 2]

Melting point: literature value: 46 – 47 °C; experimental value: 40 – 43 °C.

The compound is reported in literature.[4]

Synthesis of ethyl 4-oxo-1,4-dihydroquinoline-3-carboxylate (6)

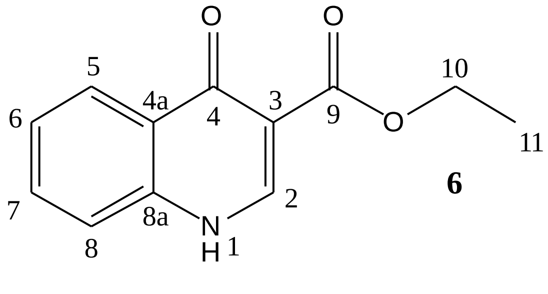

6

Ethyl 4-oxo-1,4-dihydroquinoline-3-carboxylate (**6**) was synthesized according to the general procedure.

Yield: 65%

TLC (silica plates; eluent EtOAc:DCM:Hexane 1:8:1): $R_{f\,(Substrate)}$ = 0.42; $R_{f\,(Product)}$ = 0

^{1}H NMR (400 MHz, DMSO-d_6): δ 12.29 ppm (s, 1H, H-1), 8.53 ppm (s, 1H, H-2), 8.14 ppm (dd, *J* = 8.1 Hz; 1.5 Hz, 1H, H-5), 7.70 ppm (ddd, *J* = 8.4 Hz; 7.0 Hz; 1.6 Hz, 1H, H-7), 7.60 ppm (d, *J* = 7.1 Hz, 1H, H-

8), 7.40 ppm (ddd, *J* = 8.1 Hz; 7.0 Hz; 1.2 Hz, 1H, H-6), 4.20 ppm (q, *J* = 7.1 Hz, 2H, H-10), 1.27 ppm (t, *J* = 7.1 Hz, 3H, H-11)

^{13}C NMR (101 MHz, DMSO-d_6): δ 173.4 ppm (C-4), 164.8 ppm (C-9),144.8 ppm (C-2), 138.9 ppm (C-8a), 132.4 ppm (C-7), 127.2 ppm (C-4a), 125.6 ppm (C-5), 124.6 ppm (C-6), 118.7 ppm (C-8), 109.8 ppm (C-3), 59.5 ppm (C-10), 14.3 ppm (C-11)

MS (ESI, Water); m/z (relative intensity): 240 (M + Na^+, 100%), 218 (M^+, 7%)

HR-MS (ESI, Water): 240.0631 [M + Na^+]; calculated value for $C_{12}H_{11}NNaO_3$: 240.0631 (Δ -0.1 ppm)

The compound is reported in literature.[5]

Synthesis of 4-oxo-1,4-dihydroquinoline-3-carboxylic acid (7)

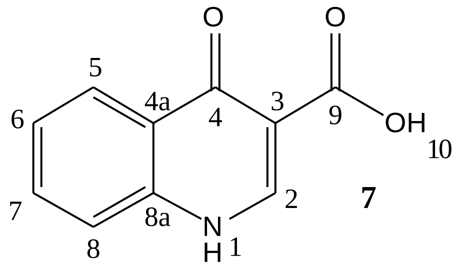

7

4-Oxo-1,4-dihydroquinoline-3-carboxylic (**7**) acid was synthesized according to the general procedure.

Yield: 87 %

TLC (silica plates; eluent EtOAc): $R_{f\,(Product)}$ = 0

^{1}H NMR (400 MHz, DMSO-d_6): δ 15.34 ppm (s, 1H, H-10), 13.42 ppm (s, 1H, H-1), 8.90 ppm (s, 1H, H-2), 8.30 ppm (dd, *J* = 8.2 Hz; 1.4 Hz, 1H, H-5), 7.89 ppm (ddd, *J* = 8.4 Hz; 6.9 Hz; 1.5 Hz, 1H, H-7), 7.82 ppm (dd, *J* = 8.5 Hz; 1.2 Hz, 1H, H-8), 7.61 ppm (ddd, *J* = 8.2 Hz; 6.9 Hz; 1.2 Hz, 1H, H-6)

^{13}C NMR (101 MHz, DMSO-d_6): δ 178.3 ppm (C-4), 166.4 ppm (C-9), 145.2 ppm (C-2), 139.5 ppm (C-8a), 133.9 ppm (C-7), 126.2 ppm (C-6), 125.0 ppm (C-5), 124.4 pp m (C-4a), 119.7 ppm (C-8), 107.6 ppm (C-3)

MS (EI, Water); m/z (relative intensity): 145 (100%), 117 (72%), 90 (39%)

HR-MS (ESI, Water): 212.0317 [M + Na^+]; calculated value for $C_{10}H_7NNaO_3$: 212.0318 (Δ 0.3 ppm)

This compound is reported in literature.[6]

Synthesis of 3-chloro-5,6-difluoro-1*H*-indole (8)

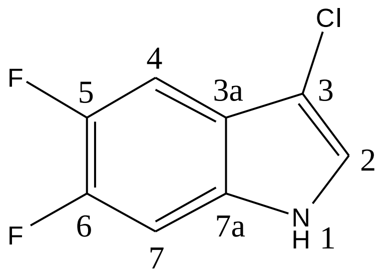

8

3-Chloro-5,6-difluoro-1*H*-indole (**8**) was synthesized according to the general procedure

Yield: 87%

TLC (silica plates; eluent pure DCM): $R_{f\,(5,6\text{-difluoroindole})}$ = 0.64 (pink spot); $R_{f\,(\text{Product})}$ = 0.64 (green spot) when developed in 5% H_2SO_4/MeOH

^{1}H NMR (400 MHz, $CDCl_3$): δ 8.04 ppm (s, 1H, H-1), 7.35 ppm (dd, *J* = 10.1 Hz; 7.6 Hz, 1H, H-4), 7.18 ppm (d, *J* = 2.6 Hz, 1H, H-2), 7.14 ppm (dd, *J* = 10.3 Hz; 6.4 Hz, 1H, H-7)

^{13}C NMR (101 MHz, $CDCl_3$): δ 148.8 ppm (dd, *J* = 243.4 Hz; 16.0 Hz, C-F, C-5), 147.1 ppm (dd, *J* = 237.4 Hz; 15.1 Hz, C-F, C-6), 129.7 ppm (d, *J* = 10.6 Hz, C, C-7a), 122.1 ppm (d, *J* = 3.9 Hz, CH, C-2), 120.9 ppm (dd, *J* = 8.6 Hz; 1.4 Hz, C, C-3a), 106.7 ppm (dd, *J* = 4.6 Hz; 1.9 Hz, C, C-3), 105.2 ppm (dd, *J* = 20.5 Hz; 1.4 Hz, CH, C-4), 99.6 ppm (d, *J* = 22.2 Hz, CH, C-7)

^{19}F NMR (376 MHz, $CDCl_3$, decoupled ^{1}H): δ -141.9 ppm (d, *J* = 20.4 Hz, F-6), -145.6 ppm (d, *J* = 20.4 Hz, F-5)

^{19}F NMR (376 MHz, $CDCl_3$, coupled ^{1}H): δ -141.9 ppm (ddd, *J* = 20.5 Hz; 10.2 Hz; 7.5 Hz, F-6), -145.6 ppm (ddd, *J* = 20.6 Hz; 10.1 Hz; 6.3 Hz, F-5)

MS (EI, Methanol); m/z (relative intensity): 187 (M^+, 100%), 189 (M^++2, 33%), 152 (31%), 125 (51 %)

HR-MS (ESI, Methanol): 186.9995 [M^+]; calculated value for $C_8H_4ClF_2N$: 186.9995 (Δ -0.3 ppm)

Melting point: 77 – 80 °C.

Synthesis of ethyl 4-chloro-6,7-difluoroquinoline-3-carboxylate (9)

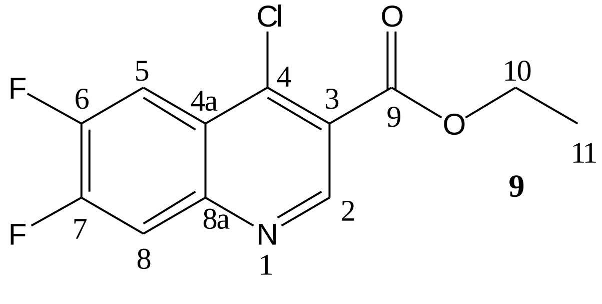

9

Ethyl 4-chloro-6,7-difluoroquinoline-3-carboxylate (**9**) was synthesized according to the general procedure.

Yield: 75% (with Cl-EDA in toluene)

TLC (silica plates; eluent EtOAc:DCM:Hexane 1:8:1): $R_{f\,(Substrate)}$ = 0.65; $R_{f\,(Product)}$ = 0.47

^{1}H NMR (400 MHz, $CDCl_3$): δ 9.15 ppm (s, 1H, H-2), 8.13 ppm (dd, *J* = 10.9 Hz; 8.3 Hz, 1H, H-5), 7.87 ppm (dd, *J* = 10.5 Hz; 7.6 Hz, 1H, H-8), 4.48 ppm (q, *J* = 7.2 Hz, 2H, H-10), 1.44 ppm (t, *J* = 7.1 Hz, 3H, H-11)

^{13}C HMR (101 MHz, $CDCl_3$): δ 164.0 ppm (s, C=O, C-9), 153.7 ppm (dd, *J* = 260.5 Hz; 16.0 Hz, C-F, C-6), 151.1 ppm (dd, *J* = 256.3 Hz; 15.7 Hz, C-F, C-7), 150.7 ppm (d, *J* = 2.9 Hz, CH, C-2), 146.9 ppm (dd, *J* = 11.2 Hz; 1.4 Hz, C, C-8a), 142.5 ppm (dd, *J* = 5.6 Hz; 2.2 Hz, C, C-4a), 123.7 ppm (d, *J* = 7.6 Hz, C, C-4), 123.2 ppm (d, *J* = 2.6 Hz, C, C-3), 116.3 ppm (dd, *J* = 17.0 Hz; 1.6 Hz, CH, C-8), 111.7 ppm (dd, *J* = 20.6 Hz; 2.0 Hz, CH, C-5), 62.3 ppm (s, CH_2, C-10), 14.2 ppm (s, CH_3, C-11)

^{19}F NMR (377 MHz, $CDCl_3$, coupled to ^{1}H): δ -127.4 ppm (ddd, *J* = 21.0 Hz; 10.7 Hz; 8.2 Hz, F-7), -131.3 ppm (ddd, *J* = 21.1 Hz; 10.9 Hz; 7.5 Hz, F-6)

^{19}F NMR (377 MHz, $CDCl_3$, decoupled to ^{1}H): δ -127.4 ppm (d, *J* = 21.0 Hz, F-7), -131.3 ppm (d, *J* = 21.1 Hz, F-6)

MS (EI, Methanol); m/z (relative intensity): 271 (M^{+}, 28%), 273 (M^{+}+2, 9%), 243 (39%), 245 (13%), 226 (100%), 228 (33%), 198 (48%), 200 (17%)

HR-MS (ESI, Methanol): 294.0104 [M + Na^{+}]; calculated value for $C_{12}H_8ClF_2NNaO_2$: 294.0104 (Δ -0.1 ppm)

Melting point: 109 – 111 °C. No literature value reported.

This compound is reported in literature.[7]

Synthesis of ethyl 6,7-difluoro-4-oxo-1,4-dihydroquinoline-3-carboxylate (10)

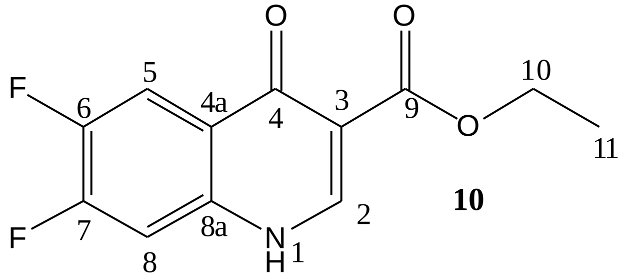

10

Ethyl 6,7-difluoro-4-oxo-1,4-dihydroquinoline-3-carboxylate (**10**) was synthesized according to the general procedure.

Yield: 90%

TLC (silica plates; eluent EtOAc:Hexane 1:1): $R_{f\,(Substrate)}$ = 0.56; $R_{f\,(Product)}$ = 0

^{1}H NMR (300 MHz, CF_3COOD): δ 9.70 ppm (s, 1H, H-2), 8.78 ppm (dd, *J* = 9.1 Hz; 7.6 Hz, 1H, H-5), 8.39 ppm (dd, *J* = 9.1 Hz; 6.3 Hz, 1H, H-8), 5.06 ppm (q, *J* = 7.2 Hz, 2H, H-10), 1.90 ppm (t, *J* = 7.2 Hz, 3H, H-11)

^{13}**C NMR** (101 MHz, CF_3COOD): δ 175.1 ppm (d, *J* = 4.2 Hz, C=O, C-4), 169.7 ppm (s, C=O, C-9), 160.1 ppm (dd, *J* = 271.7 Hz; 15.8 Hz, C-F, C-6), 154.8 ppm (dd, *J* = 263.6 Hz; 14.5 Hz, C-F, C-7), 148.1 ppm (s, CH, C-2), 139.9 ppm (d, *J* = 11.7 Hz, C, C-8a), 120,0 ppm (dd, *J* = 8.2 Hz; 1.7 Hz, C, C-4a), 114.5 ppm (dd, *J* = 20.9 Hz; 2.9 Hz, CH, C-5), 111.0 ppm (d, *J* = 22.5 Hz, CH, C-8), 107.7 ppm (s, C, C-3), 67.5 ppm (s, CH_2, C-10), 14.6 ppm (s, CH_3, C-11)

^{19}**F NMR** (377 MHz, CF_3COOD, coupled to ^{1}H): δ -114.1 ppm (dd, *J* = 18.7 Hz; 8.9 Hz, F-6), -126.6 ppm (dt, *J* = 16.8 Hz; 7.6 Hz, F-7)

^{19}**F NMR** (377 MHz, CF_3COOD, coupled to ^{1}H): δ -114.1 ppm (d, *J* = 19.2 Hz, F-6), -126.6 ppm (d, *J* = 19.5 Hz, F-7)

MS (EI, Methanol); m/z (relative intensity): 253 (M^+, 30%), 208 (17%), 107 (100%), 180 (14%)

HR-MS (ESI, Methanol): 276.0443 [M + Na^+]; calculated value for $C_{12}H_9F_2NNaO_3$: 276.0443 (Δ -0.2 ppm)

Melting point: The compound decomposes at 255 °C. No literature value reported.

This compound is reported in literature.[8]

Synthesis of ethyl 1-ethyl-6,7-difluoro-4-oxo-1,4-dihydroquinoline-3-carboxylate[9]

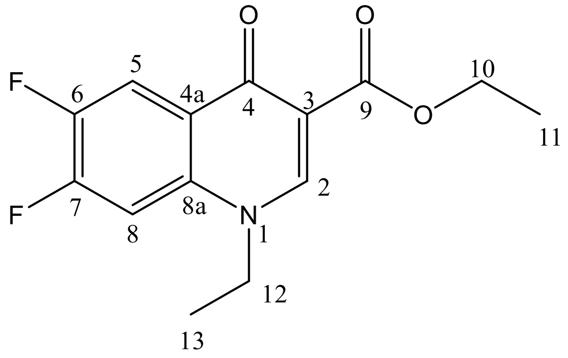

Ethyl 6,7-difluoro-4-oxo-1,4-dihydroquinoline-3-carboxylate (501.6 mg; 1.98 mmol; 1.0 equiv), K_2CO_3 (429.7 mg; 3.11 mmol; 1.6 equiv) and CH_3CH_2I (210 µL; 2.63 mmol; 1.33 equiv) were suspended in 7 mL dry DMF and the solution was heated at 90 °C for 24 h. The solution prior to heating was foggy white, after reflux transparent. 10 mL brine and 4 mL H_2O were added to the cold solution and the product was extracted with 4 x 10 mL EtOAc. The collected organic phases were washed with 4 x 10 mL brine and 2 x 10 mL H_2O, dried with Na_2SO_4 and evaporated in vacuo. The product was purified with flash chromatography on a silica plug with gradient elution from EtOAc:hexane (1:1) to pure EtOAc. This gave a white solid, 472.7 mg (1.68 mmol).

Yield: 85%

TLC (silica plates; eluent pure EtOAc): $R_{f\ (Substrate)}$ = 0; $R_{f\ (Product)}$ = 0.21

^{1}**H NMR** (400 MHz, $CDCl_3$): δ 8.47 ppm (s, 1H, H-2), 8.30 ppm (dd, *J* = 10.5 Hz; 8.8 Hz, 1H, H-5), 7.24 ppm (dd, *J* = 10.4 Hz; 6.3 Hz, 1H, H-8), 4.38 ppm (q, *J* = 7.1 Hz, 2H, H-10), 4.18 ppm (q, *J* = 7.3 Hz, 2H, H-12), 1.53 ppm (t, *J* = 7.3 Hz, 3H, H-13), 1.39 ppm (t, *J* = 7.1 Hz, 3H, H-11)

^{13}C NMR (101 MHz, $CDCl_3$): δ 172.6 ppm (s, C=O, C-4), 165.5 ppm (s, C=O, C-9), 153.5 ppm (dd, *J* = 256 Hz; 15.3 Hz, C-F, C-7), 148.4 ppm (dd, *J* = 252 Hz; 13.3 Hz, C-F, C-6), 148.7 ppm (s, CH, C-2), 135.6 ppm (dd, *J* = 9.2 Hz; 1.9 Hz, C, C-8a), 126.7 ppm (dd, *J* = 4.8 Hz; 2.1 Hz, C, C-4a), 115.9 ppm (dd, *J* = 18.6 Hz; 2.5 Hz, CH, C-5), 111.2 ppm (s, C, C-3), 104.5 ppm (d, *J* = 22.5 Hz, CH, C-8), 61.1 ppm (s, CH_2, C-10), 49.3 ppm (s, CH_2, C-12), 14.4 ppm (s, CH_3, C-11), 14.3 ppm (s, CH_3, C-13)

^{19}F NMR (376 MHz, $CDCl_3$, coupled to ^{1}H): δ (-127.3) – (-127.5) ppm (m, F-7), -138.7 ppm (ddd, *J* = 22.2 Hz; 10.3 Hz; 6.1 Hz, F-6)

^{19}F NMR (376 MHz, $CDCl_3$, decoupled to ^{1}H): δ -127.4 ppm (d, *J* = 22.2 Hz, F-7), -138.7 ppm (d, *J* = 22.2 Hz, F-6)

MS (EI, Acetonitrile); m/z (relative intensity): 281 (M^+, 6%), 236 (23%), 209 (100%), 208 (14%), 194 (34%)

HR-MS (ESI, Acetonitrile): 304.0755 [M + Na^+]; calculated value for $C_{14}H_{13}F_2NNaO_3$: 304.0767 (Δ 0.2 ppm)

Melting point: Literature value: 154 - 155 °C; Experimental value: 149 - 153 °C

This compound is reported in literature.[10]

Synthesis of ethyl 1-ethyl-6-fluoro-4-oxo-7-(piperazin-1-yl)-1,4-dihydroquinoline-3-carboxylate from piperazine and ethyl 6,7-difluoro-4-oxo-1,4-dihydroquinoline-3-carboxylate

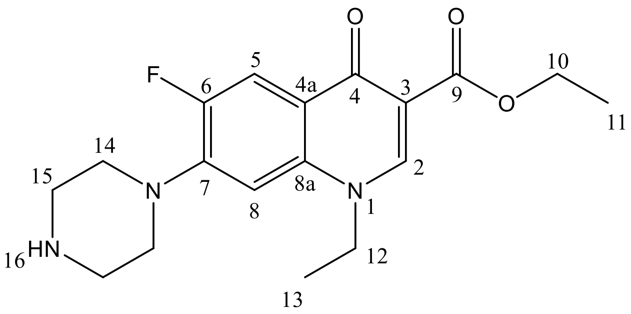

Ethyl 1-ethyl-6,7-difluoro-4-oxo-1,4-dihydroquinoline-3-carboxylate (42.9 mg; 0.15 mmol; 1.0 equiv) and piperazine (60.6 mg; 0.70 mmol; 4.6 equiv) were dissolved in 1.0 mL MeCN, which gave an orange solution. The reaction was refluxed overnight (27 h), the solution cooled and the solvent was evaporated in vacuo to gave an off-white solid. Both ^{1}H and ^{19}F NMR analysis indicated full conversion. Even though this reaction provided the desired target molecule, we found the purification and isolation difficult due to solubility and chromatographic complications. Hence, the yield was measured by internal standard (IS) using ^{1}H NMR.

Yield: 98% (IS)

TLC (silica plates; eluent pure EtOAc): $R_{f\ (Substrate)}$ = 0.21; $R_{f\ (Product)}$ = 0

^{1}H NMR (400 MHz, $CDCl_3$): δ 8.25 ppm (s, 1H, H-2), 7.88 ppm (d, *J* = 13.4 Hz, 1H, H-5), 6.61 ppm (d, *J* = 6.9 Hz, 1H, H-8), 4.25 ppm (q, *J* = 7.1 Hz, 2H, H-10), 4.09 ppm (q, *J* = 7.2 Hz, 2H, H-12), 3.10 ppm (dd, *J* = 6.3Hz; 3.4 Hz, 4H, H-14), 2.97 ppm (dd, *J* = 6.3 Hz; 3.5 Hz, 4H, H-15), 1.90 ppm (s, 1H, H-16), 1.42 ppm (t, *J* = 7.2 Hz, 3H, H-13), 1.29 ppm (t, *J* = 7.1 Hz, 3H, H-11)

^{13}C NMR (101 MHz, $CDCl_3$): δ 172.8 ppm (d, *J* = 2.2 Hz, C=O, C-4), 165.4 ppm (s, C=O, C-9), 152.9 ppm (d, *J* = 248.7 Hz, C-F, C-6), 147.6 ppm (s, CH, C-2), 144.9 ppm (d, *J* = 10.6 Hz, C, C-7), 135.8 ppm (d, *J* = 1.3 Hz, C, C-8a), 123.3 ppm (d, *J* = 6.9 Hz, C, C-4a), 113.1 ppm (d, *J* = 23.1 Hz, CH, C-5), 109.9 ppm (s, C, C-3), 103.6 ppm (d, *J* = 3.1 Hz, CH, C-8), 60.5 ppm (s, CH_2, C-10), 51.0 ppm (d, *J* = 4.5 Hz, CH_2, C-14), 48.8 ppm (s, CH_2, C-12), 45.7 ppm (s, CH_2, C-15), 14.2 ppm (s, CH_3, C-11), 14.1 ppm (s, CH_3, C-13)

^{19}F NMR (376 MHz, $CDCl_3$, coupled to ^{1}H): δ -123.5 ppm (dd, *J* = 13.5 Hz; 6.8 Hz, F-6)

^{19}F NMR (376 MHz, $CDCl_3$, decoupled to ^{1}H): δ -123.5 ppm (s, F-6)

MS (EI, Water); m/z (relative intensity): 347 (M^+, 13%), 305 (100%), 302 (6%), 275 (47%)

HR-MS (ESI, Water): 348.1718 [M + H^+]; calculated value for $C_{18}H_{23}FN_3O_3$: 348.1718 (Δ 0.1 ppm)

The compound is reported in literature.[11]

Synthesis of ethyl 7-(4-(*tert*-butoxycarbonyl)piperazin-1-yl)-1-ethyl-6-fluoro-4-oxo-1,4-dihydroquinoline-3-carboxylate (11)

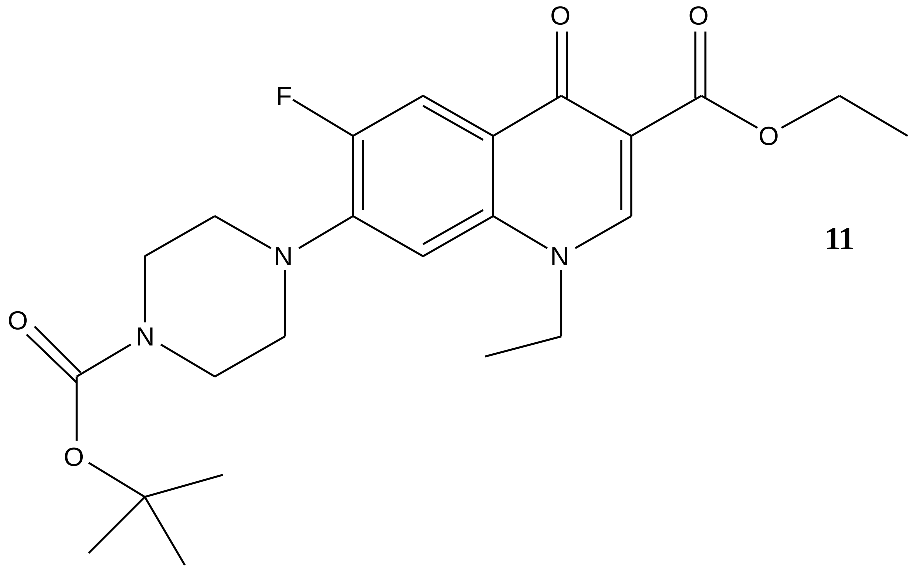

11

Ethyl 6,7-difluoro-4-oxo-1,4-dihydroquinoline-3-carboxylate (101.5 mg; 0.36 mmol; 1.0 equiv), Boc-piperazine (599.0 mg; 3.22 mmol; 8.9 equiv) and K_2CO_3 (153.1 mg; 1.11 mmol; 3.1 equiv) were dissolved in 2.0 mL MeCN and heated to reflux for 3 days. The solvent was evaporated in vacuo and ^{1}H NMR analysis of the crude products indicated over 95% conversion. The crude mixture was dissolved in 10 mL EtOAc and extracted with water (pH 4–5) until all Boc-piperazine was removed. The residue was purified with flash chromatography on a silica plug with gradient elution from pure EtOAc until 10% MeOH concentration. This gave **11** as an off-white solid 113.8 mg (0.25 mmol).

Yield: 70%

TLC (silica plates; eluent MeOH:EtOAc 1:20): $R_{f\,(Substrate)}$ = 0.40; $R_{f\,(Product)}$ = 0.27

^{1}H NMR (400 MHz, Acetone-d_6): δ 8.54 ppm (s, 1H, H-2), 7.90 ppm (d, *J* = 13.4 Hz, 1H, H-5), 7.17 ppm (d, *J* = 7.3 Hz, 1H, H-8), 4.47 ppm (q, *J* = 7.2 Hz, 2H, H-10), 4.24 ppm (q, *J* = 7.1 Hz, 2H, H-12), 3.61 ppm

(t, *J* = 5.1 Hz, 4H, H-14), 3.25 ppm (t, *J* = 5.1 Hz, 4H, H-15), 1.52 ppm (t, *J* = 7.2 Hz, 3H, H-11), 1.47 ppm (s, 9H, H-18) 1.30 ppm (t, *J* = 7.1 Hz, 3H, H-13)

^{13}C NMR (101 MHz, Acetone-d_6): δ 172.5 ppm (d, *J* = 2.0 Hz, C=O, C-4), 165.7 ppm (s, C=O, C-9), 153.8 ppm (d, *J* = 247.1 Hz, C-F, C-6), 154.9 ppm (s, C=O, C-16), 149.2 ppm (s, CH, C-2), 145.3 ppm (d, *J* = 10.6 Hz, C, C-7), 137.3 ppm (s, C, C-8a), 124.5 ppm (dd, *J* = 6.2 Hz; 1.7 Hz, C, C-4a), 113.1 ppm (d, *J* = 22.8 Hz, CH, C-5), 111.0 ppm (s, C, C-3), 106.6 ppm (d, *J* = 2.2 Hz, CH, C-8), 79.9 ppm (s, C, C-17), 60.4 ppm (s, CH_2, C-12), 50.8 ppm (d, *J* = 4.0 Hz, CH_2, C-15), 49.3 ppm (s, CH_2, C-10), 44.3 ppm (d, *J* = 97.0 Hz, CH_2, C-14), 28.5 ppm (s, CH_3, C-18), 14.7 ppm (s, CH_3, C-11), 14.6 ppm (s, CH_3, C-13)

^{19}F NMR (377 MHz, Acetone-d_6, coupled to ^{1}H): δ -126.3 ppm (dd, *J* = 13.5 Hz; 7.2 Hz, F-6)

^{19}F NMR (377 MHz, Acetone-d_6, decoupled to ^{1}H): δ -126.3 ppm (s, F-6)

MS (EI, Methanol); m/z (relative intensity): 347 (13 %), 346 (1 %), 305 (100 %), 302 (6%), 275 (47 %)

HR-MS (ESI, Methanol): 470.2061 [*M* + Na^+]; calculated value for $C_{23}H_{30}FN_3NaO_5$: 470.2062 (Δ 0.1 ppm)

Melting point: Experimental value: 149-153 °C.

This compound is not reported in literature.

Synthesis of 7-(4-(*tert*-butoxycarbonyl)piperazin-1-yl)-1-ethyl-6-fluoro-4-oxo-1,4-dihydroquinoline-3-carboxylic acid

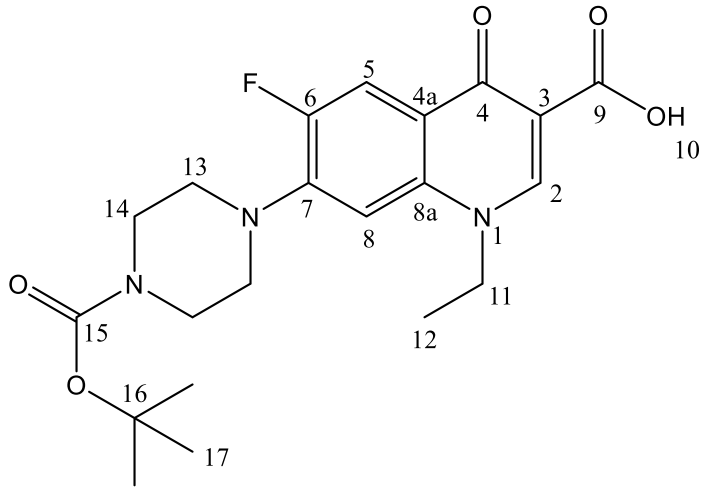

7-(4-(*tert*-Butoxycarbonyl)piperazin-1-yl)-1-ethyl-6-fluoro-4-oxo-1,4-dihydroquinoline-3-carboxylic acid was synthesized according to the general procedure. The ester substrate **11** (39.3 mg; 0.09 mmol; 1.0 equiv) and LiOH·H_2O (14.1 mg; 0.34 mmol; 3.8 equiv) in MeOH/H_2O gave 35.4 mg (0.08 mmol) of the carboxylic acid as white/yellow solid.

Yield: 96%

TLC (silica plates; eluent MeOH:EtOAc 1:20): $R_{f\ (Substrate)}$ = 0.33 $R_{f\ (Product)}$ = 0.09

^{1}H NMR (500 MHz, $CDCl_3$): δ 14.99 ppm (s, 1H, H-10), 8.67 ppm (s, 1H, H-2), 8.09 ppm (d, *J* = 12.9 Hz, 1H, H-5), 6.82 ppm (d, *J* = 6.9 Hz, 1H, H-8), 4.30 ppm (q, *J* = 7.3 Hz, 2H, H-11), 3.65 ppm (t, *J* = 4.9 Hz, 4H, H-13), 3.25 ppm (t, *J* = 5.1 Hz, 4H, H-14), 1.58 ppm (t, *J* = 7.3 Hz, 3H, H-12), 1.48 ppm (s, 9H, H-17)

^{13}C NMR (101 MHz, $CDCl_3$): δ 176.9 ppm (s, C=O, C-4), 167.1 ppm (s, C=O, C-9), 153.5 ppm (d, *J* = 252.4 Hz, C-F, C-6), 154.5 ppm (s, C=O, C-15), 147.2 ppm (s, CH, C-2), 145.9 ppm (d, *J* = 10.6 Hz, C, C-7), 137.0 ppm (s, C, C-8a), 120.9 ppm (d, *J* = 7.8 Hz, C, C-4a), 112.9 ppm (d, *J* = 23.2 Hz, CH, C-5), 108.4 ppm (s, C, C-3), 104.0 ppm (d, *J* = 3.1 Hz, CH, C-8), 80.3 ppm (s, C, C-16), 49.8 ppm (s, CH_2, C-11 and C-14), 43.3 ppm (d, *J* = 82.0 Hz, CH_2, C-13), 28.4 ppm (s, CH_3, C-17), 14.5 ppm (s, CH_3, C-12)

^{19}F NMR (376 MHz, $CDCl_3$, coupled to ^{1}H): δ -120.9 ppm (dd, *J* = 12.9 Hz; 6.8 Hz, F-6)

^{19}F NMR (376 MHz, $CDCl_3$, decoupled to ^{1}H): δ -120.9 ppm (s, F-6)

MS (ESI, Methanol); m/z (relative intensity): 456 (100%), 442 (*M* + Na^+, 98%), 413 (40%),

185 (10%)

HR-MS (ESI, Methanol): 442.1749 [*M* + Na^+]; calculated value for $C_{21}H_{26}FN_3NaO_5$:

442.1749 (Δ = -0.0 ppm)

This compound is not reported in literature.

Synthesis of 4-(3-carboxy-1-ethyl-6-fluoro-4-oxo-1,4-dihydroquinolin-7-yl)piperazin-1-ium chloride – norfloxacin hydrochloride (12)

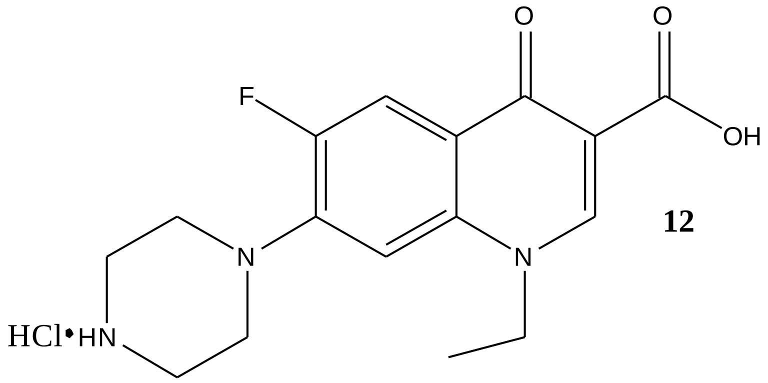

12

7-(4-(tert-Butoxycarbonyl)piperazin-1-yl)-1-ethyl-6-fluoro-4-oxo-1,4-dihydroquinoline-3-carboxylic acid (23.1 mg; 0.05 mmol; 1.0 equiv) was dissolved in 2.5 mL dry DCM and gave a foggy pale-yellow solution. TFA (0.2 mL; 2.61 mmol; 47.49 equiv) was added to the solution and gave immediate color change to yellow. The reaction was stirred overnight at ambient temperature and the solvent removed *in vacuo*. Adding a small amount of methanol-d_4 for NMR analysis gave white/brown precipitation. ^{19}F NMR analysis of the precipitate solution indicated full conversion to the trifluoroacetate salt of norfloxacin. It showed two ^{19}F NMR peaks – one peak belonging to F-6 and one to trifluoroacetate counter ion. To the crude product dissolved in 2 mL H_2O, was added 2 mL 1 M HCl. The solvent was thereafter evaporated in vacuo. This was done a second time to ensure that most of the TFA was removed from the sample. This gave **12** as a white/yellow solid, 18.2 mg (0.05 mmol). ^{1}H and ^{19}F NMR analysis indicated full conversion.

Yield: 99%

TLC (silica plates; eluent EtOAc): $R_{f\,(Product)}$ = 0

^{1}**H NMR** (400 MHz, DMSO-*d*$_{6}$): δ 15.26 ppm (bs, 1H, H-10), 9.68 ppm (bs, 1H, H-15), 8.96 ppm (s, 1H, H-2), 7.94 ppm (d, *J* = 13.1 Hz, 1H, H-5), 7.26 ppm (d, *J* = 7.0 Hz, 1H, H-8), 4.62 ppm (q, *J* = 6.5 Hz, 1H, H-11), 3.58 ppm (s, 2H, H-13), 3.28 ppm (s, 2H, H-14), 1.41 ppm (t, *J* = 6.8 Hz, 3H, H-12)

^{13}**C NMR** (101 MHz, DMSO-*d*$_{6}$): δ 176.1 ppm (s, C=O, C-4), 165.9 ppm (s, C=O, C-9), 152.7 ppm (d, *J* = 250.1 Hz, C-F, C-6), 148.7 ppm (s, CH, C-2), 144.4 ppm (d, *J* = 10.0 Hz, C, C-7), 137.1 ppm (s, C, C-8a), 119.9 ppm (s, C, C-4a), 111.4 ppm (d, *J* = 22.7 Hz, CH, C-5), 107.1 ppm (s, C, C-3), 106.5 ppm (s, CH, C-8), 49.1 ppm (s, CH_2, C-11), 46.4 ppm (s, CH_2, C-13), 42.4 ppm (s, CH_2, C-14), 14.5 ppm (s, CH_3, C-12)

^{19}**F NMR** (376 MHz, DMSO-*d*$_{6}$, coupled ^{1}H): δ -121.7 ppm (dd, *J* = 13.2 Hz; 7.0 Hz, F-6)

^{19}**F NMR** (376 MHz, DMSO-*d*$_{6}$, decoupled ^{1}H): δ -121.7 ppm (s, F-6)

MS (ESI, Water); m/z (relative intensity): 320 (M^+, 100%), 301 (6%), 293 (10%), 250 (15%), 234 (7%)

HR-MS (ESI, Water): 320.1405 [M^+]; calculated value for $C_{16}H_{19}FN_3O_3$: 320.1405 (Δ = 0.0 ppm)

This compound is reported in the literature[12] and as its' non-salt form.[13]

NMR spectral data

Ethyl 4-chloroquinoline-3-carboxylate (**4**), 400 MHz, $CDCl_3$.

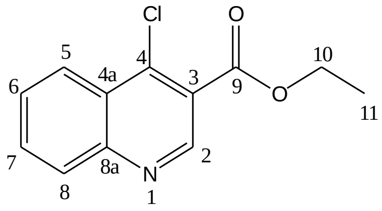

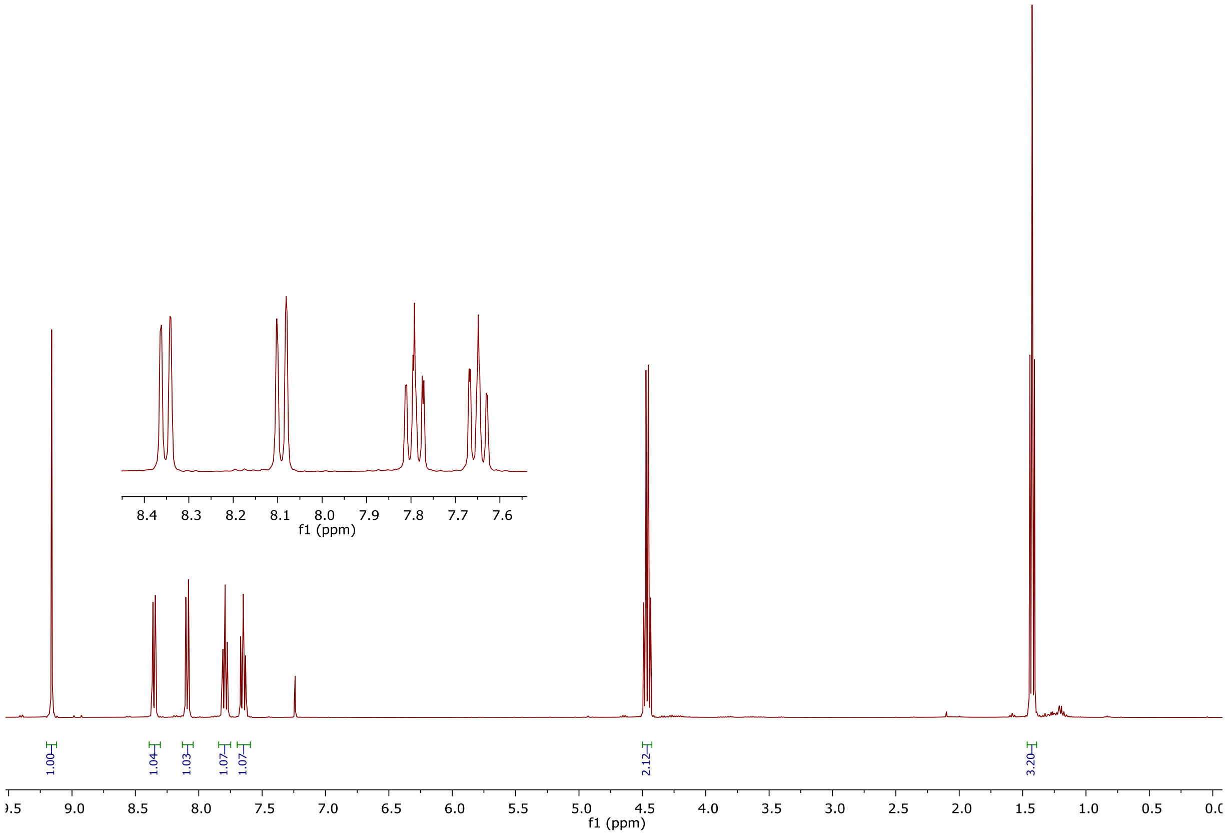

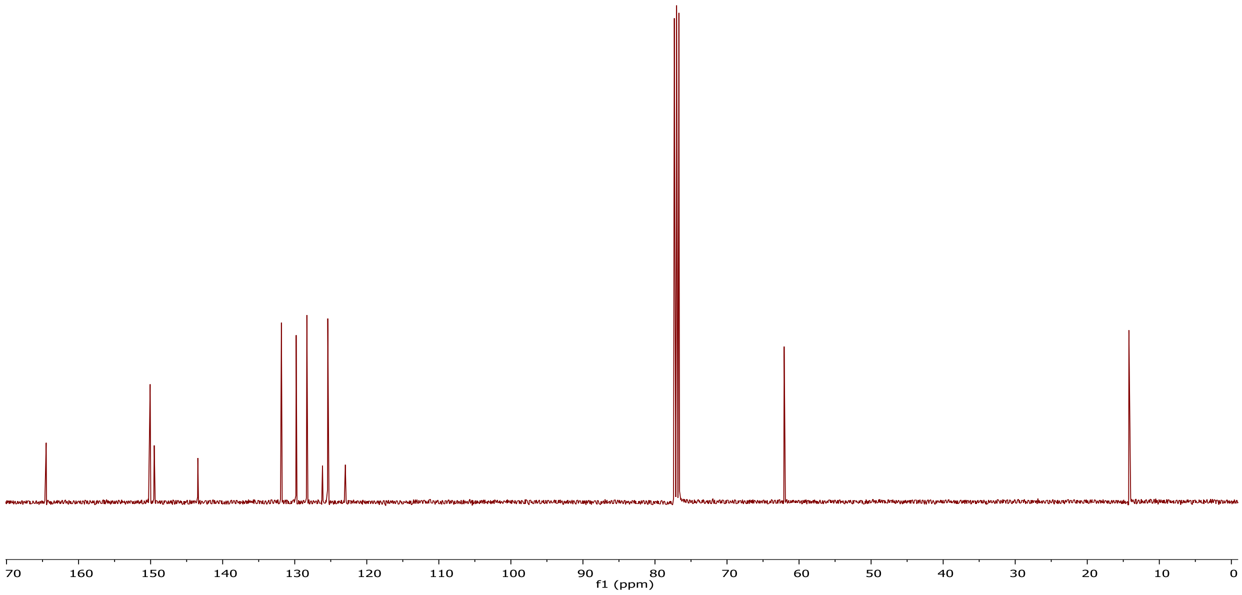

Ethyl-4-quinolone-3-carboxylate (**6**), 400 MHz, DMSO-d_6.

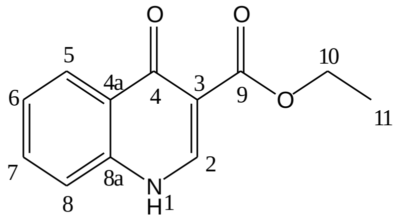

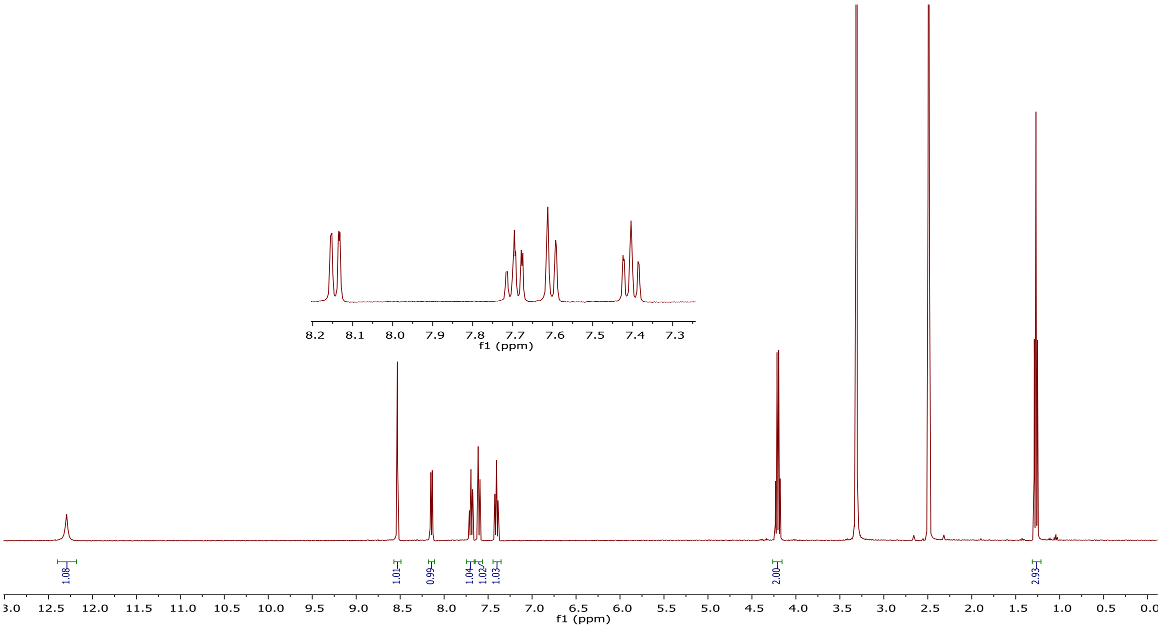

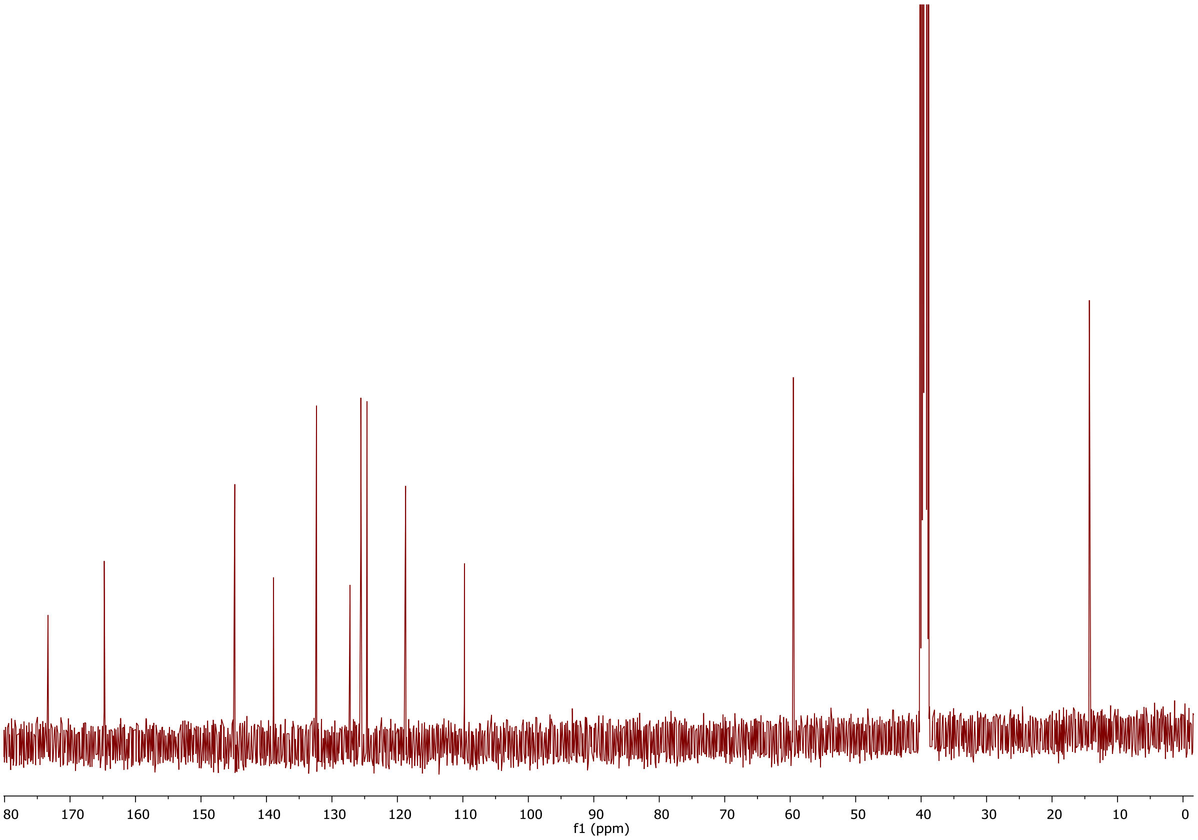

4-quinolone-3-carboxylic acid (**7**), 400 MHz, DMSO-d_6.

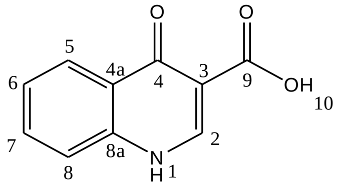

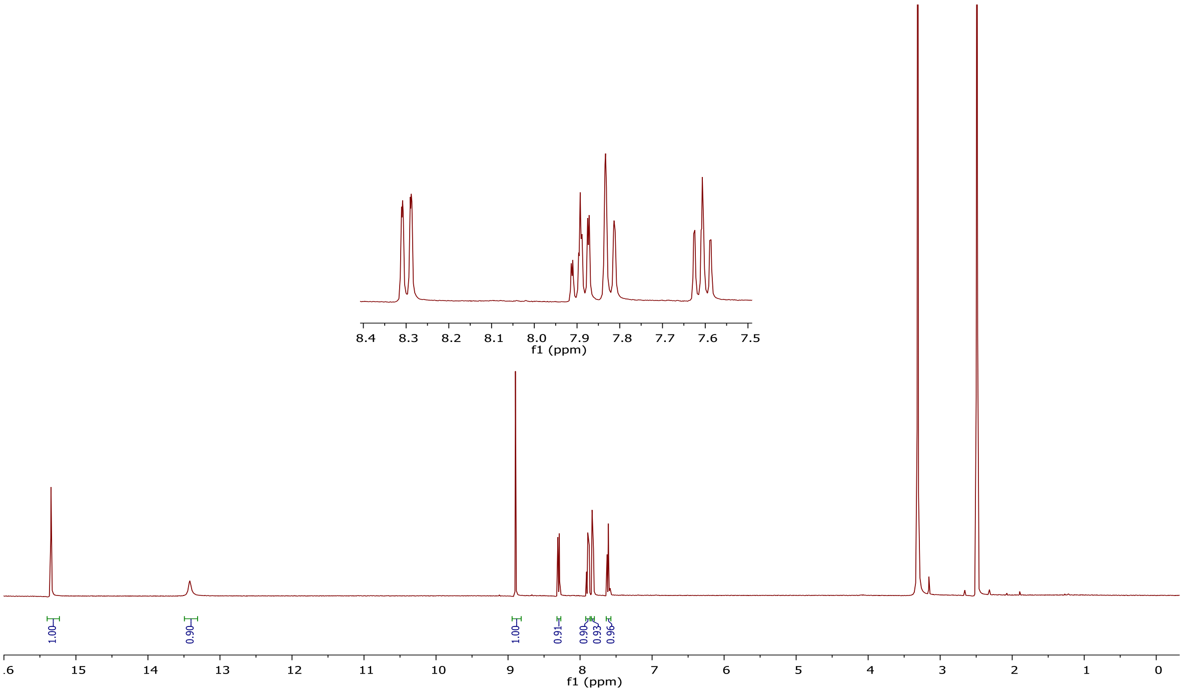

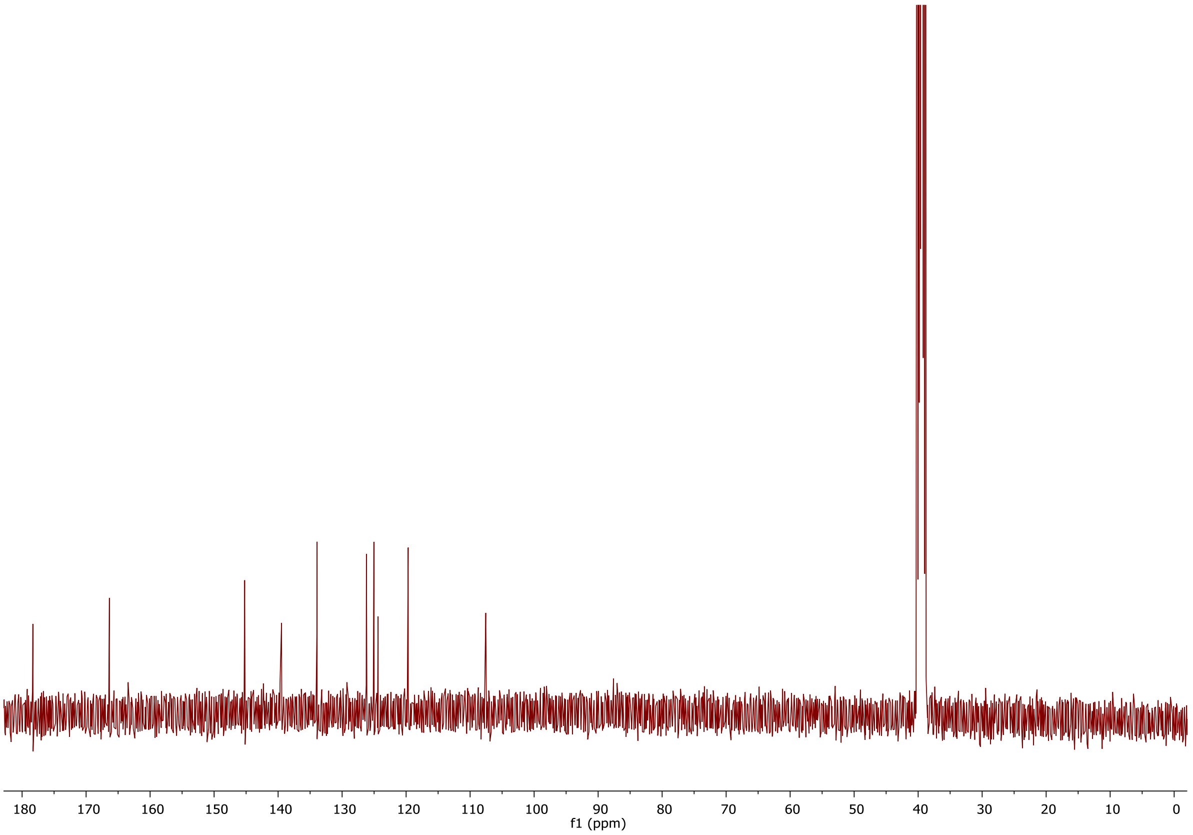

3-chloro-5,6-difluoro-1*H*-indole (**8**), 400 MHz, $CDCl_3$.

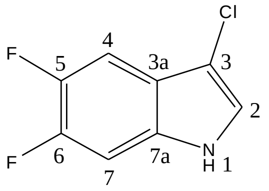

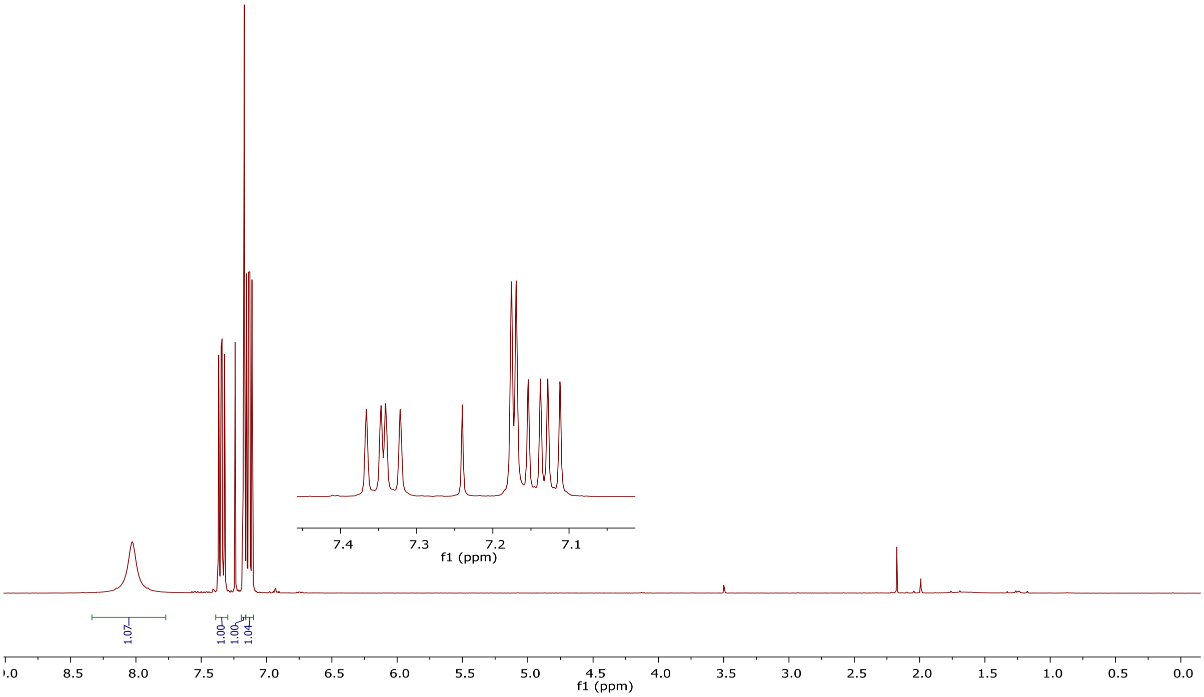

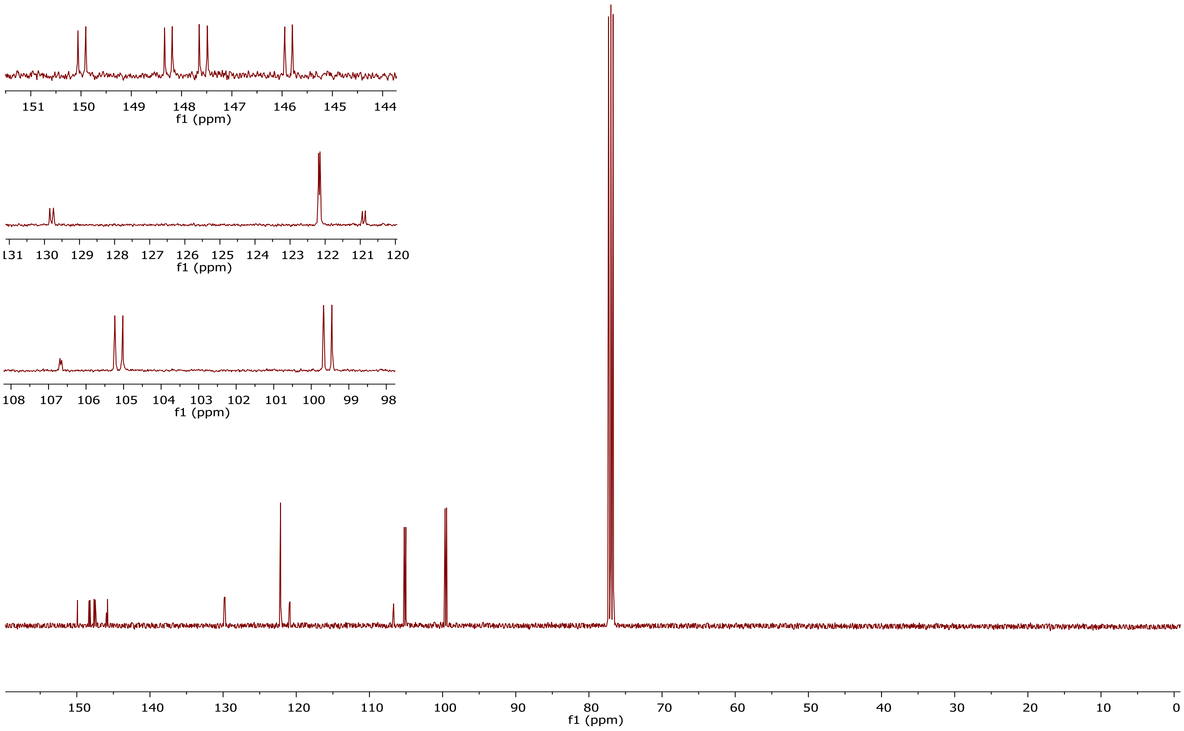

Ethyl 4-chloro-6,7-difluoroquinoline-3-carboxylate (**9**), 400 MHz, $CDCl_3$.

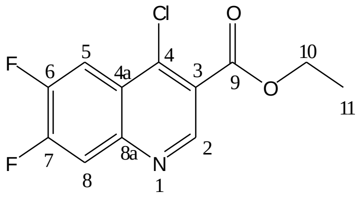

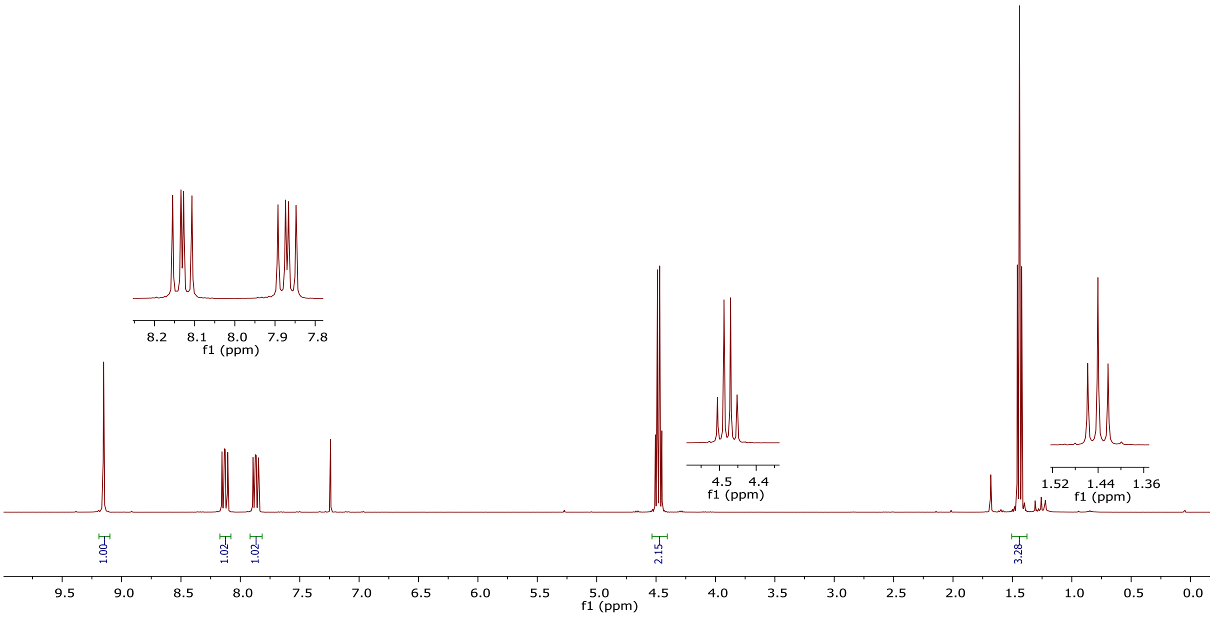

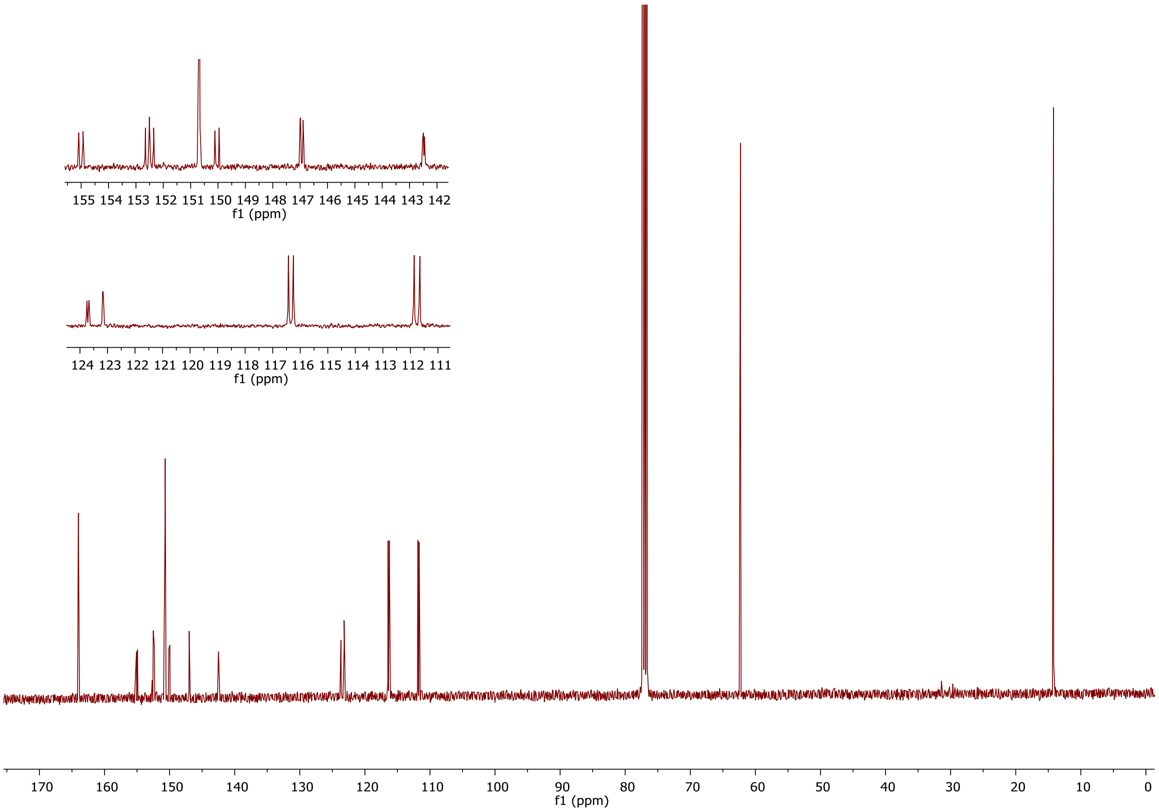

Ethyl 6,7-difluoro-4-oxo-1,4-dihydroquinoline-3-carboxylate (**10**), 300 MHz, CF_3COOD.

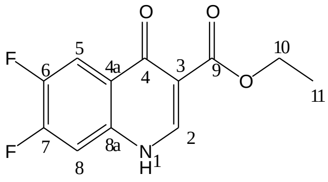

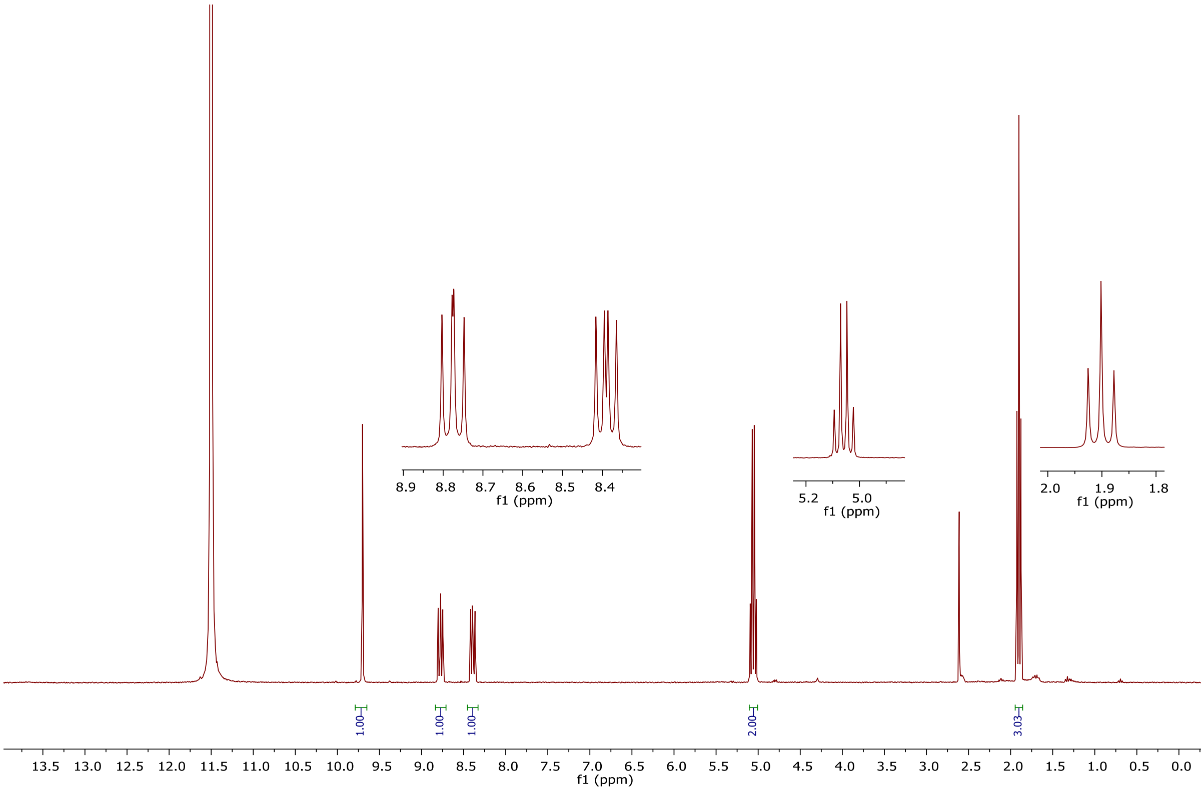

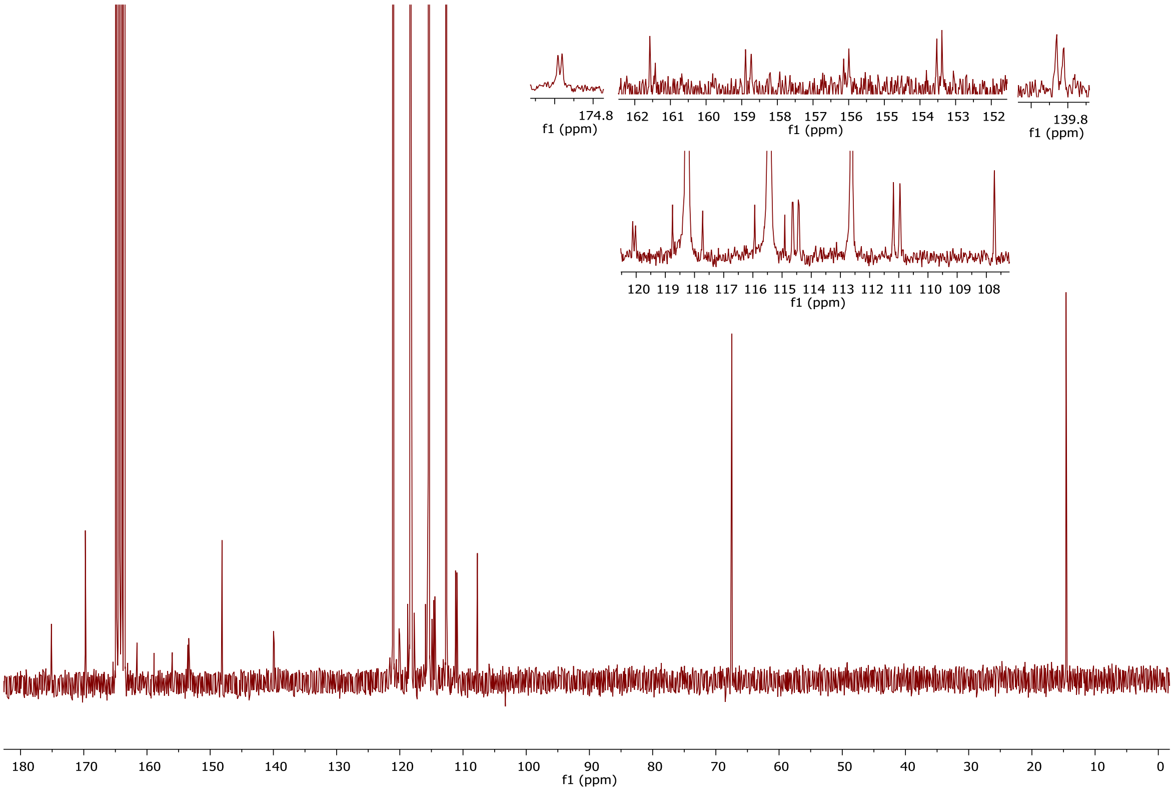

Ethyl 1-ethyl-6,7-difluoro-4-oxo-1,4-dihydroquinoline-3-carboxylate, 400 MHz, $CDCl_3$.

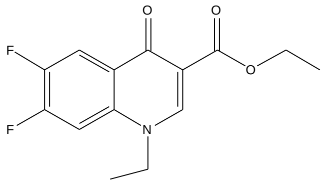

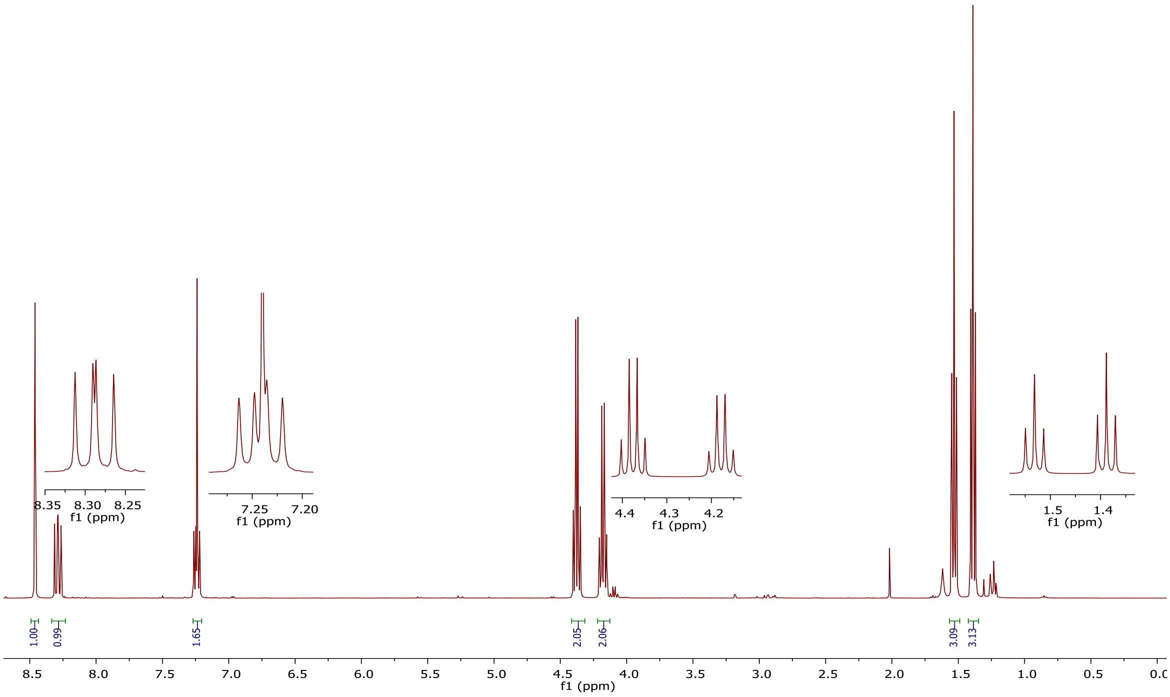

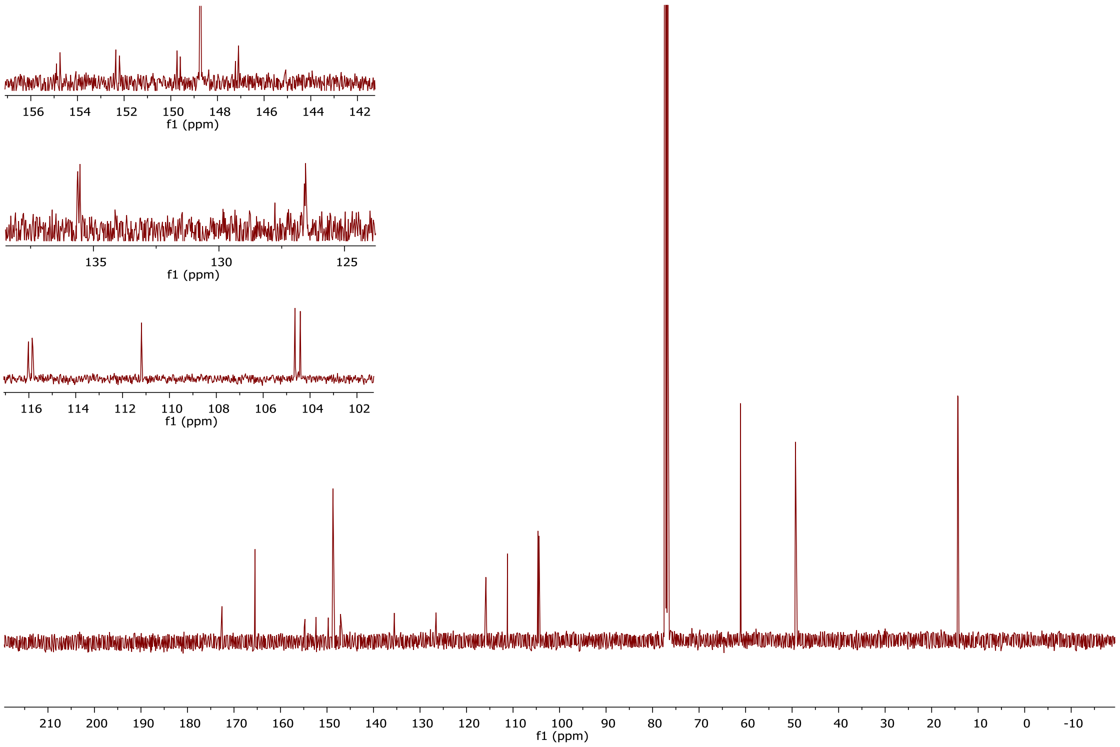

Ethyl 1-ethyl-6-fluoro-4-oxo-7-(piperazin-1-yl)-1,4-dihydroquinoline-3-carboxylate, 400MHz, $CDCl_3$.

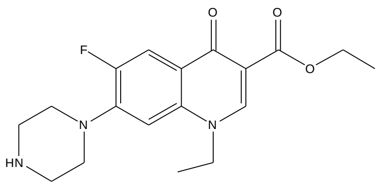

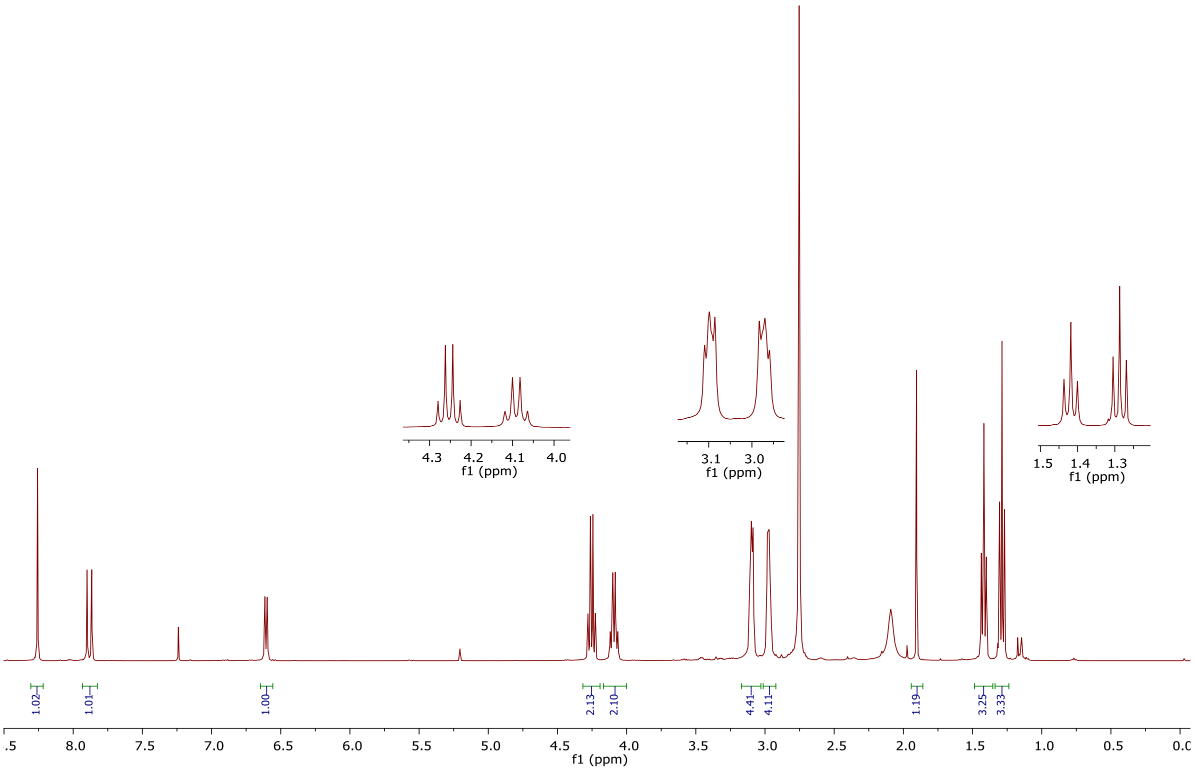

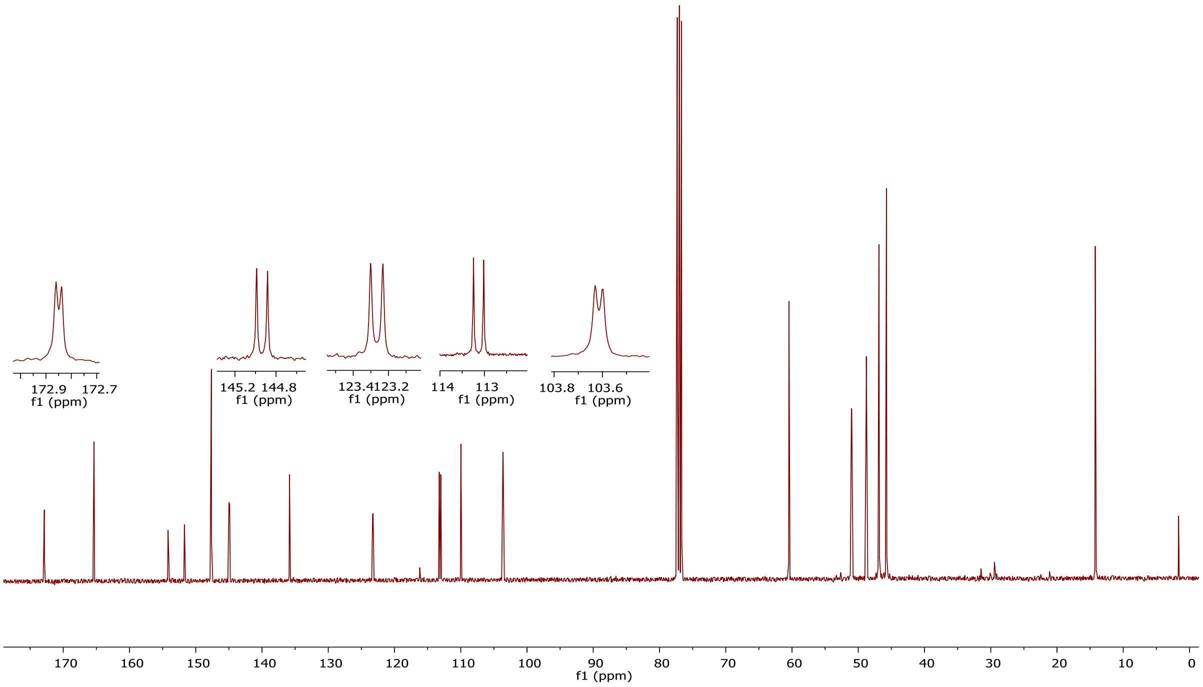

Ethyl 7-(4-(*tert*-butoxycarbonyl)piperazin-1-yl)-1-ethyl-6-fluoro-4-oxo-1,4-dihydroquinoline-3-carboxylate (**11**), 400MHz, acetone-d_6.

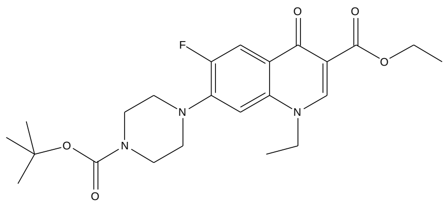

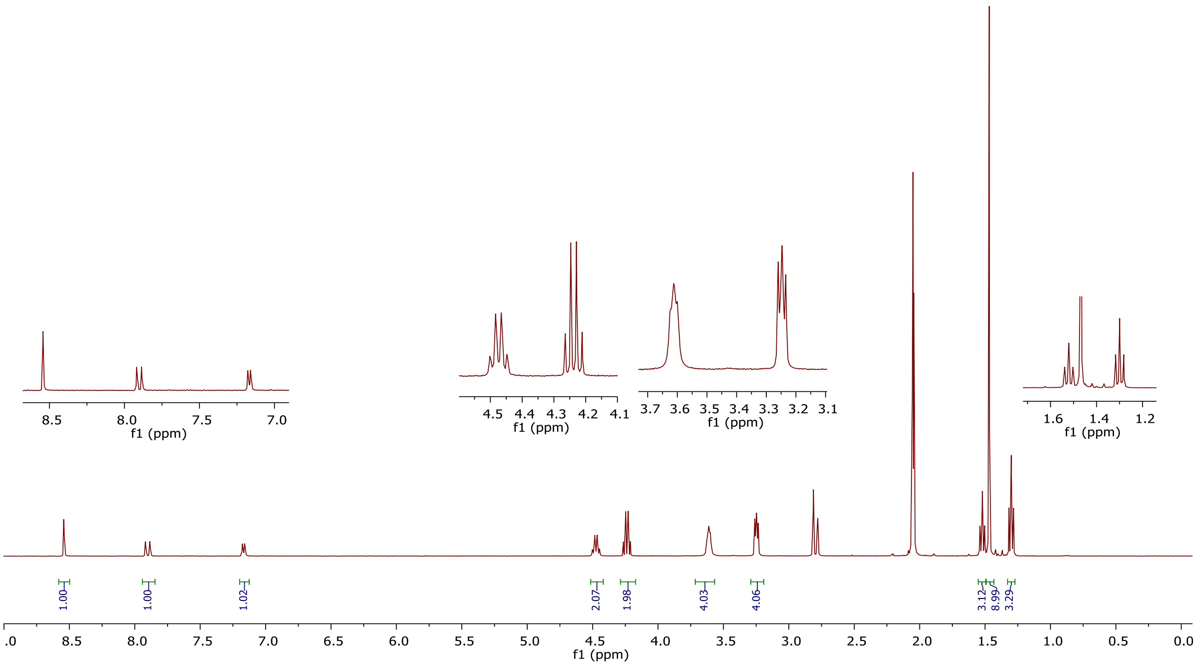

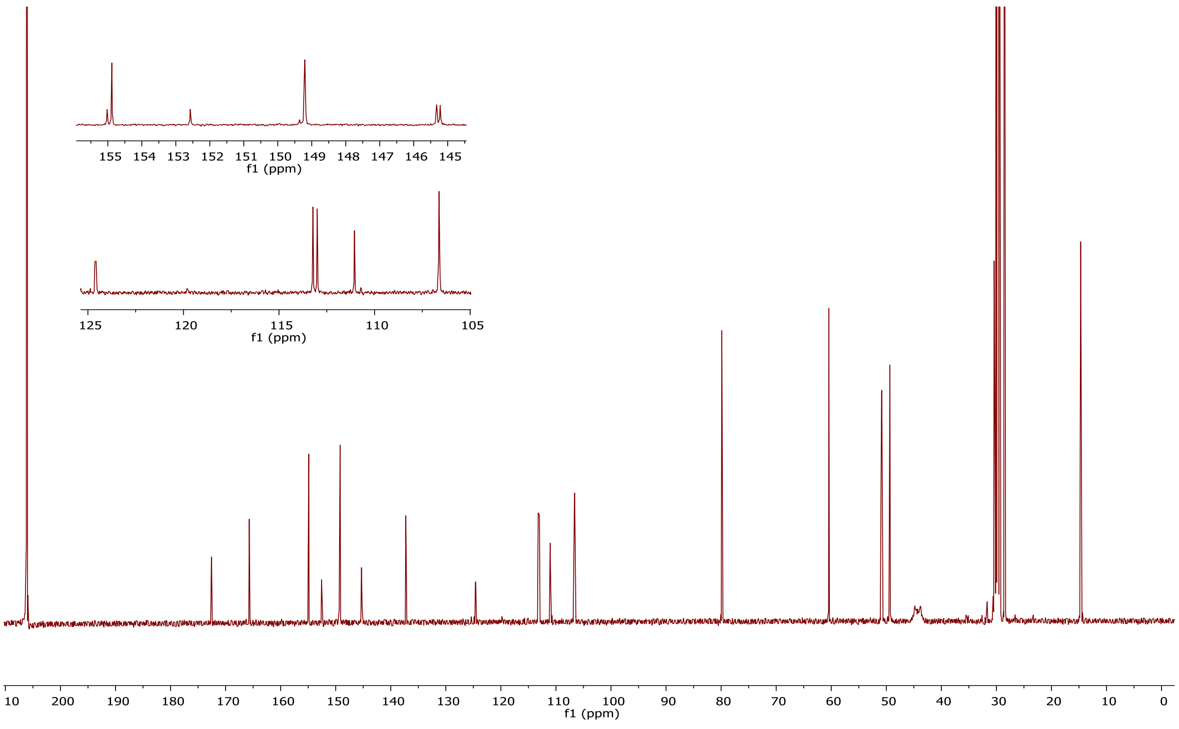

7-(4-(*tert*-Butoxycarbonyl)piperazin-1-yl)-1-ethyl-6-fluoro-4-oxo-1,4-dihydroquinoline-3-carboxylic acid, 500 MHz, $CDCl_3$.

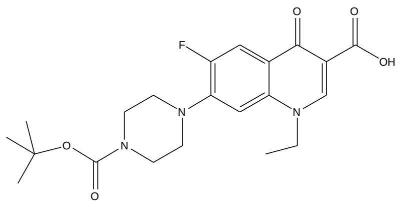

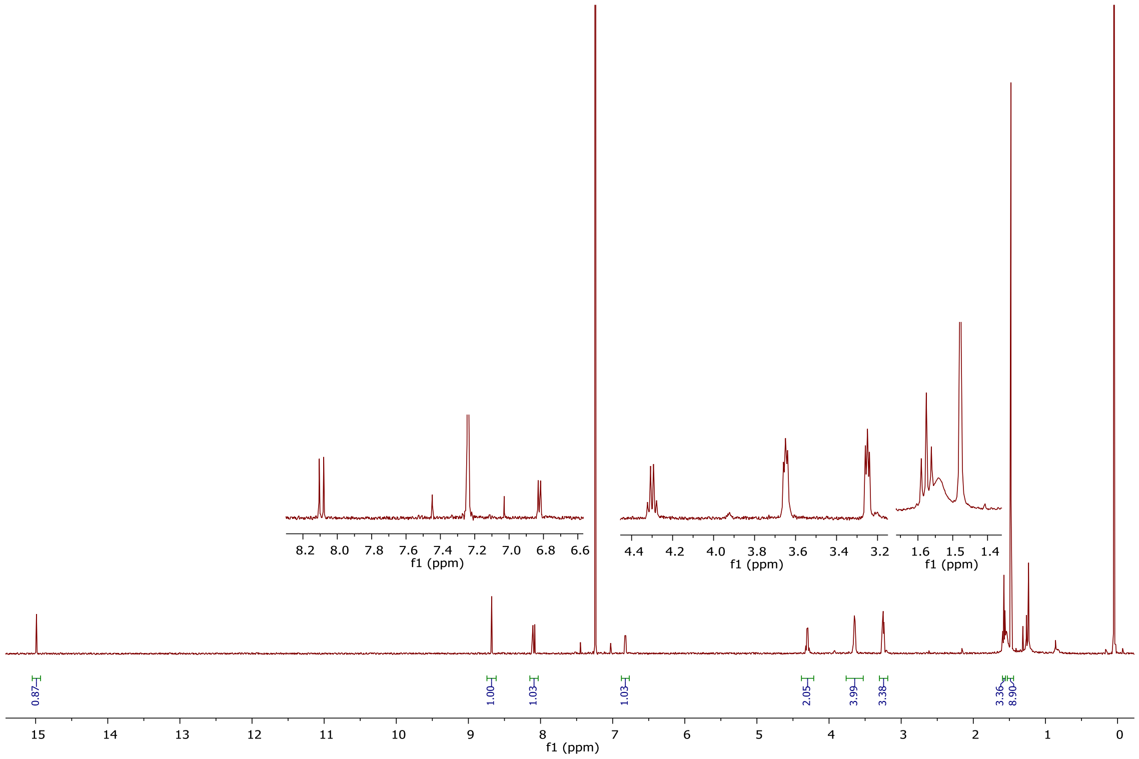

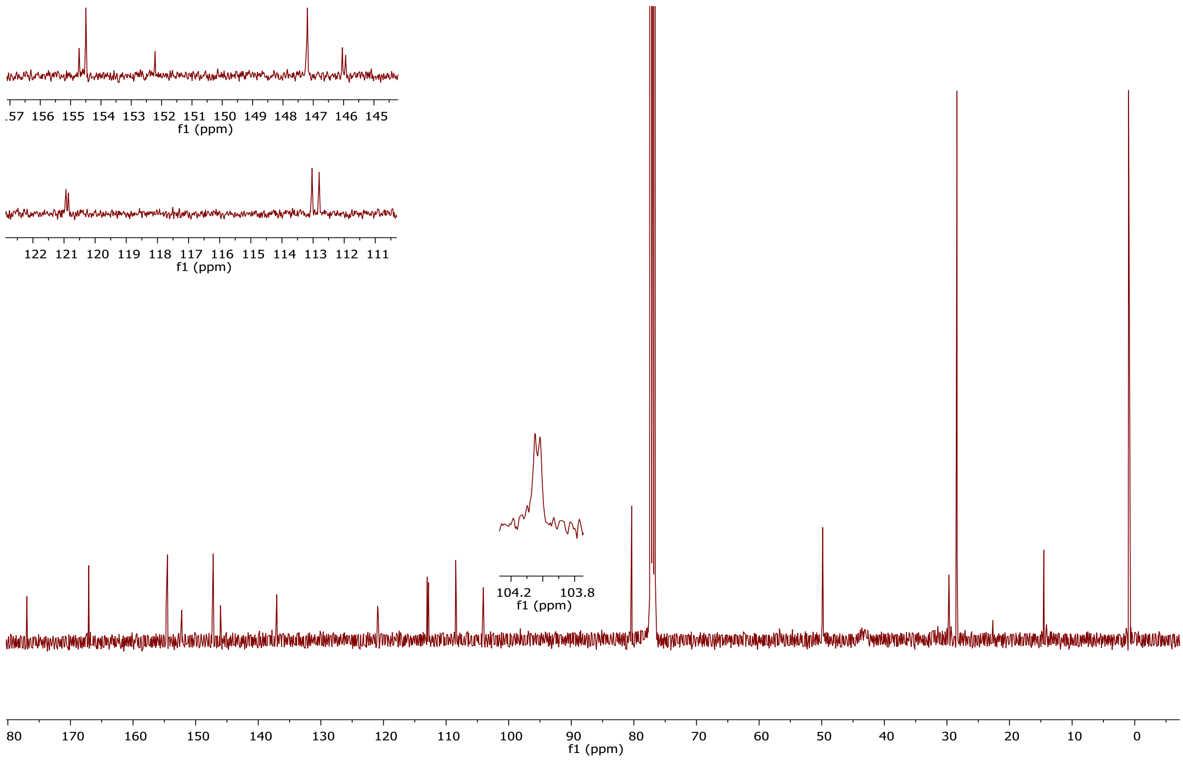

4-(3-carboxy-1-ethyl-6-fluoro-4-oxo-1,4-dihydroquinolin-7-yl)piperazin-1-ium chloride – norfloxacin hydrochloride (**12**), 400 MHz, DMSO-d_6.

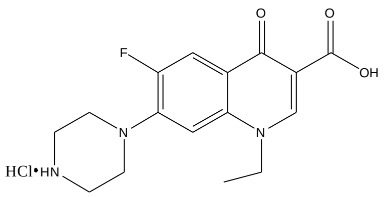
O
O
F
OH
N
N
HCl•HN

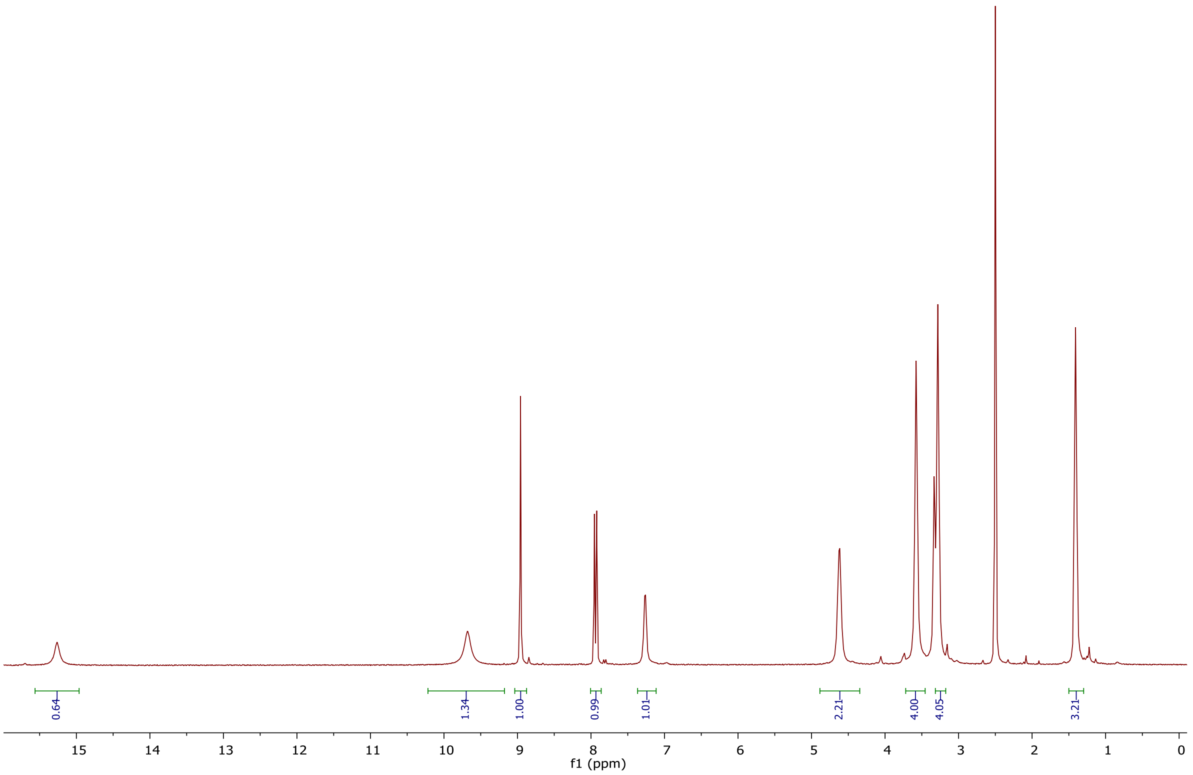
0.64
1.34
1.00
0.99
1.01
2.21
4.00
4.05
3.21
15 14 13 12 11 10 9 8 7 6 5 4 3 2 1 0
f1 (ppm)

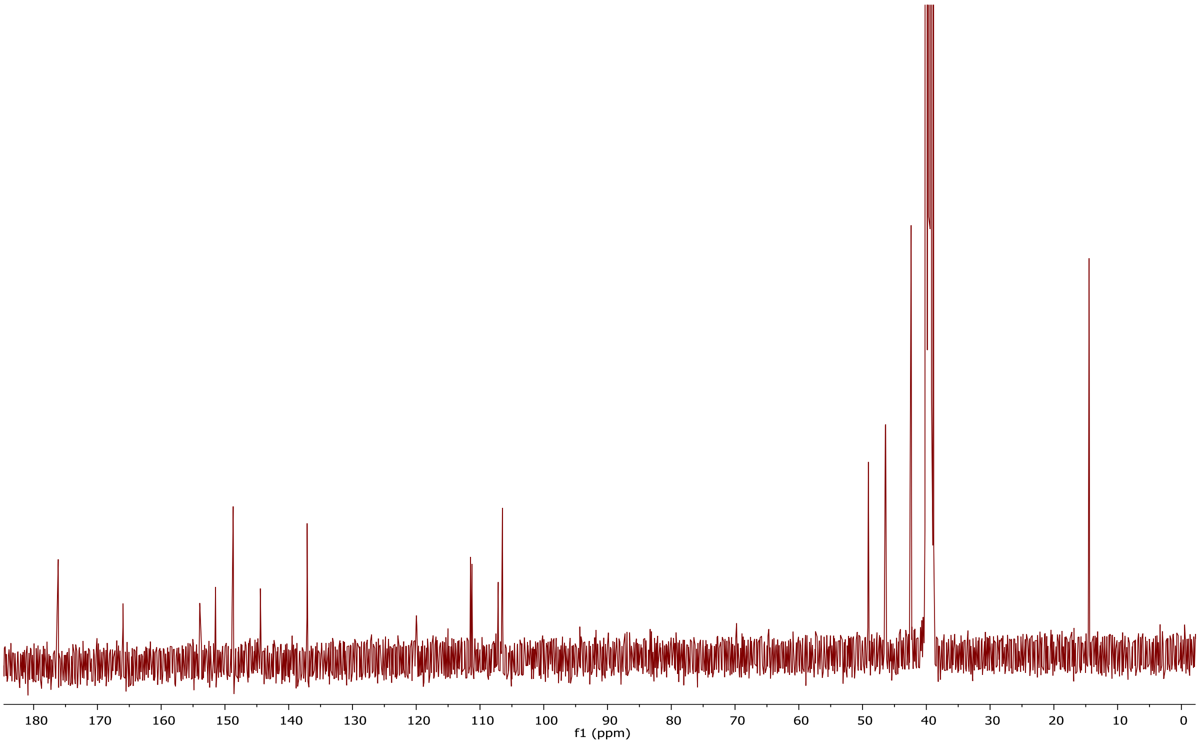
180 170 160 150 140 130 120 110 100 90 80 70 60 50 40 30 20 10 0
f1 (ppm)

References

1. H. T. Bonge, B. Pintea, T. Hansen, *Org. Biomol. Chem.* 2008**, 6**, 3670.
2. C. A. Kuttruff, H. Zipse, D. Trauner, *Angew. Chem., Int. Ed.* 2011**, 50**, 1402. A.C05, S1402/1-S1402/45.
3. N. D. Heindel, S. A. Fine, *J. Org. Chem*. 1970, **35**, 796.
4. T. Takahashi, S. Senda, *Yakugaku Zasshi* 1952**, 72**, 1112.
5. C. M. Hall, H. G. Johnson, J. B. Wright, *J. Med. Chem.* 1974**, 17**, 685.
6. M. F. Grundon, H. J. McCorkindale, M. N. Rodger, *J. Chem. Soc.* 1955, 4284.
7. Kaplan, A. P.; Gupta, V. Preparation of pyrazoloquinolonecarboxamides as GABAA receptor modulators. US20080306048A1, 2008.
8. Brighty, K. E.; Lowe, J. A., III; McGuirk, P. R. Preparation of 6-fluoro-1,4-dihydro-4-oxo-(1,8-naphthyridine or quinoline)-3-carboxylic acid derivatives as antibacterial agents. EP321191A2, 1989.
9. V. Venepally, R.B.N. Prasad, Y. Poornachandra, C. G. Kumar, R. C. R. Jala, *Bioorg. Med. Chem. Lett.* 2016**, 26**, 613.
10. B. Podanyi, G. Kereszturi, L. Vasvaridebreczy, I. Hermecz, G. Toth, *Magn. Reson. Chem.* 1996**, 34**, 972.
11. H. Koga, A. Itoh, S. Murayama, S. Suzue, T. Irikura, *J. Med. Chem.* 1980**, 23**, 1358.
12. Jose Gorrindo, P.; Martinez Morato, V.; Dominguez Buron, E.; Oranias Olsina, G. Piperazinoquinolinecarboxylic acids. ES540055A1, 1985.
13. Irikura, T.; Suzue, S.; Ito, A.; Koga, H. Quinolinecarboxylic acid derivative. JP54138582A, 1979.
